# Can natural scenes cue attention to multiple locations? Evidence from eye-movements in contextual cueing

**DOI:** 10.3389/fcogn.2024.1352656

**Published:** 2024-03-13

**Authors:** Josefine Albert, Werner X. Schneider, Christian H. Poth

**Affiliations:** ^1^Neuro-Cognitive Psychology, Department of Psychology, Bielefeld University, Bielefeld, Germany; ^2^Center for Cognitive Interaction Technology, Bielefeld University, Bielefeld, Germany

**Keywords:** attention, contextual cueing, natural scenes, multiple target locations, learning, visual search, reaction time

## Abstract

Humans find visual targets more quickly when the target appears at the same location in a repeated configuration of other stimuli. However, when the target alternates between two locations in the repeated configuration, the benefit for visual search is smaller. This reduction of benefits has been explained as the result of an averaging of a benefit for one location and a cost for the other location. In two experiments, we investigated this two-target-locations effect in real-world scenes using high-resolution eye-tracking. Experiment 1 adapted a study in which subjects searched for a small “T” or “L” superimposed on real-world photographs. Half of the trials showed repeated scenes with one possible target location each; half showed novel scenes. We replicated the pronounced contextual cueing effect in real-world scenes. In Experiment 2, two conditions were added. In one of them, targets appeared in repeated scenes alternating between two possible locations per scene. In the other condition, targets appeared in repeated scenes but at new locations, constrained to one side of the screen. Subjects were faster to search for and identify a target in repeated scenes than in novel scenes, including when the scene was paired with two alternating target locations and (after extensive training) even when the scene only predicted the hemifield. Separate analyses on the two possible target locations resulted in rejection of the suggestion of costs for the additional target location, since the contextual cueing effect was present in the second half of the experiment for both the favored and the less favored target location. The eye-tracking data demonstrated that contextual cueing influences searching fixations, characteristic of attentional guidance, rather than responding fixations, characteristic of facilitation of response processes. Further, these data revealed that adding another possible target location leads to less guidance, rather than impeding response processes. Thus, this study delivers evidence for a flexible and attentional guidance mechanism that is able to prioritize more than one location in natural contexts.

## 1 Introduction

The present study asks whether humans can develop a search benefit through incidental learning that allows them to find a target more quickly in the context of a specific, repeatedly occurring natural scene than in unknown contexts, even when the target is not always in the same place, but is always in one of two possible places.

When humans encounter specific scenes repeatedly, e.g., their offices, they may learn that certain stimuli are more likely to be at specific locations within those scenes, e.g., that the wastepaper bin is behind the second desk. While being abundant in visual information, at the same time, real-world scenes bear regularities that humans can make use of in principle. On the one hand, general scene information helps observers to recognize expected object categories and to search for objects adaptively contingent on the association between scene type and object type (e.g., Biederman et al., 1982; Torralba et al., 2006; Ehinger et al., 2009; Võ and Henderson, 2009). On the other hand, regularities within specific scenes could also be exploited (episodic guidance, cf. Wolfe et al., 2011). Indeed, research has shown that repeatedly searching for and identifying an object in a context where the target object consistently appears at one specific location speeds up search and discrimination compared to searching for the object in similar, not repeatedly encountered contexts. This effect, i.e., the benefit to response time in repeated vs. novel contexts, has been termed the contextual cueing effect (Chun and Jiang, 1998) and has been replicated many times (for reviews, see Goujon et al., 2015; Sisk et al., 2019). The contexts used in the seminal study by Chun and Jiang (1998) and many of the subsequent studies were not real-world scenes but configurations of L-shaped distractors in which participants searched for a T-shaped target. Participants reported the orientation of the target more quickly in configurations that were repeated throughout the experiment (“repeated contexts”) compared to configurations generated randomly for a particular trial and shown only once (“novel contexts”). The difference between these two conditions was not only the repetition of the repeated contexts and the novelty of the new contexts. In addition, in each repeated context, the target appeared consistently at only one possible location. Despite their ability to use this contextual information, in a later, unannounced memory test, participants could not explicitly discriminate repeated contexts from new contexts.

Here, we ask whether contexts can facilitate visual search for only a single target location. So far, evidence is mixed and stems exclusively from experiments using artificial displays (e.g., Chun and Jiang, 1998; Beesley et al., 2015, Experiment 6; Kunar et al., 2005; Kunar and Wolfe, 2011, Experiment 4; Zellin et al., 2011; Wang et al., 2020; Vadillo et al., 2021). Chun and Jiang (1998) observed a contextual cueing effect for old configurations that were associated with two target locations. The experiment lasted for 20 blocks. In ten randomly selected blocks, each old configuration was paired with one location and, in the other ten blocks, it was paired with the second location. In epochs 4 and 5 (blocks 13 to 20), participants responded more quickly to targets in old configurations than to targets in new configurations. However, this significant contextual cueing effect was smaller [35 ms] than in their Experiment 1 [66 ms], where old configurations were associated with only one consistent target location. Additionally, Kunar et al. (2005) reported that a context could cue two target locations to a descriptively smaller extent, and even four target locations with extended training, provided that no distractor ever appeared in a target location. Similarly, in a study by Zellin et al. (2011), repeated configurations were paired with two target locations shown alternately every block of trials. This resulted in a contextual cueing effect. Again, the contextual cueing effect was smaller than for contexts paired with only one location [67 ms vs. 169 ms]. However, when the authors paired contexts with three target locations, a contextual cueing effect no longer arose. Zellin et al. (2011) suggested that the smaller contextual cueing effect in contexts associated with two target locations and the absence of a contextual cueing effect for contexts associated with three target locations resulted from averaging a positive contextual cueing effect for one dominant target location with a low or even negative contextual cueing effect for the other minor location(s). They calculated the contextual cueing effect separately for the two or three locations associated with the repeated contexts. Then they sorted them into “dominant” and “minor” locations with larger and smaller contextual cueing effects, respectively. The size of the contextual cueing effect for the dominant locations was similar to that of a control selection of dominant contexts in the condition with only one associated target location. However, the size of the contextual cueing effect for the minor locations (one of the minor locations for the three-target contexts) was smaller than zero and different from that of the minor control selection. The authors took this as suggesting that only a single target location is effectively cued by a particular context. Moreover, they concluded that the negative contextual cueing effect for the minor location represents contextual costs. This term might implicate an attentional suppression of the minor target location. However, what is meant is that having learnt the dominant location for a certain context would result in attention being guided toward it, and consequently guided away from the minor target location, leading to costs when the target appears at the unexpected (not learnt) minor target location. Consistent with this view, the authors observed that contextual cueing effects were smaller/more negative for the minor target locations the further away they were from the dominant target location. The costs were therefore not specific to the minor target location. From a priority map point of view (e.g., Wolfe, 1994; Fecteau and Munoz, 2006; Zelinsky and Bisley, 2015), having learnt to prioritize one location in a context would mean a bump (high priority) in the priority map at this location. If, in a next step, the relative priority of all locations is calculated, this would necessarily turn out smaller (than the baseline without learning) for all non-learned locations, including the minor location(s) through normalization. Thus, it is not necessary to assume a dip in priority (below-baseline priority) specifically for the minor target location(s). This understanding of the results on contextual cueing effects for multiple target locations reviewed above is compatible with an attentional guiding account of contextual cueing, i.e., the assumption that the response time benefit is due to more efficient guiding to specific locations in the context, which is based on associative connections between the (visual details and/or identity of the) context and locations within the context (see, e.g., Chun and Jiang, 1998; Brady and Chun, 2007; Harris and Remington, 2020; cf. Guided Search, Wolfe, 2021).

In contrast, Wang et al. (2020) put forward the hypothesis that attentional guidance cannot be the only mechanism driving the contextual cueing effect in their study. They also studied contextual cueing with multiple target locations and observed significant contextual cueing when every two or every four (but not every twelve) repeated distractor configurations switched their target locations randomly across blocks. The aspect of the findings that was inconsistent with an attentional guidance account was that the contextual cueing effect they observed for contexts associated with two target locations was comparable in size to the one observed for contexts associated with four target locations. Following the logic that guidance toward one location necessarily is misguiding in at least half of the cases when there is more than one possible location, they concluded that there must be another mechanism facilitating search in all cases. Furthermore, they stated that the contextual cueing effect in contexts associated with two target locations was likely comparable between the two targets and differences between dominant and minor locations could largely be explained by eccentricity effects. Wang et al. (2020) propose that facilitation of decision or response selection processes might, in contrast, be the mechanism that was responsible for the comparable contextual cueing effects in two- and four-target-location contexts.

We drew several questions from this conflicting evidence. First, we wondered, since all of the studies so far have used arbitrary configurations as contexts, whether learning and prioritizing of multiple target locations might be simpler in more natural contexts, and whether their use might produce clearer evidence for a contextual cueing effect. From observation of everyday life, we establish the notion that there are several possible object locations that are especially relevant for any given context, and that we efficiently orient ourselves to any of these locations dependent on the circumstances. It is still unclear whether multiple target locations can be cued by natural scenes. Contextual cueing has been shown to be very effective when natural or naturalistic scenes are used as contextual cues (e.g., Brockmole and Henderson, 2006a,b; Rosenbaum and Jiang, 2013; Pollmann et al., 2020). In a study by Brockmole and Henderson (2006b), participants searched for a small letter superimposed on real-world photographs and indicated whether the letter was “T” or “L.” Half of the trials showed repeated scenes with one possible target location each; half showed novel scenes. The resulting contextual cueing effect was large, in the range of seconds. This peculiarity of very large effects might help us to demonstrate the presence or absence of contextual cueing effects for multiple target locations more clearly. Mainly, however, by using natural scenes as contextual cues we intend to move the question closer toward everyday life situations. Still, we are aware that natural scenes as stimuli might be qualitatively different from configurational contexts with regard to contextual cueing. One noteworthy difference is that the repetition of configurations usually goes unnoticed by participants, and repeated and novel configurations cannot be discriminated. Despite this, it is a matter of debate as to whether the inability to find evidence for explicit awareness of repeated displays in array-based contextual cueing paradigms might not be due to the absence of explicit memory for repeated contexts (and their associated target locations), but rather attributable to a lack of power on the part of the typically short recognition tests (Vadillo et al., 2020). Nevertheless, configurational contextual cueing is commonly considered an example of implicit learning. Conversely, participants readily recognize repeated natural scene contexts as old (e.g., Brockmole and Henderson, 2006b). We therefore also measured scene recognition to clarify whether this was the case in our study as well. Other differences for natural scene contexts as compared to configurational contexts include the relatively early onset of learning (e.g., Brockmole and Henderson, 2006b), dependency on rather global than local contexts (Brooks et al., 2010; Castelhano et al., 2019), and the time course of the unfolding the effect in a trial (Peterson and Kramer, 2001; Brockmole and Henderson, 2006a; Summerfield et al., 2006).

Our second question pertains to the mechanism driving contextual cueing effects for multiple target locations. As already mentioned above, two main “loci” of contextual cueing are commonly discussed. Recent reviews have concluded that repeating contexts with consistent target locations in a visual search task probably affects two phases of the visual search process, namely, a first phase of attentional guidance (early locus) and a second phase of facilitation of response-related processes (late locus; Goujon et al., 2015; Sisk et al., 2019). Manual response times blend these phases and therefore are unsuitable to distinguish them without drawing on sophisticated behavioral manipulations. By contrast, studying eye movement behavior allows for direct measurement of these two phases of the visual search process. For example, eye-tracking makes it possible to divide the search process in two phases: an initial guidance phase before the target is fixated, and a second response-selection phase from the first target fixation until the manual response. In this way, eye-tracking studies have often found an effect of contextual cueing on the first phase, but in some cases on both phases (cf. Sisk et al., 2019). Notably, these studies have been conducted for artificial configurations only (e.g., Zhao et al., 2012; Harris and Remington, 2017). Here, we applied similar logic; however, not only did we measure the temporal length of the phases, but we also treated the fixations of the early and late phases of the search behavior as two classes of fixations. We measured the two phases of the search process by distinguishing and analyzing searching vs. responding fixations (see also, Epelboim et al., 1995; Foerster and Schneider, 2015). Concretely, we considered all fixations that are made following the last time the gaze enters the area around the target to be responding fixations that are made in the preparation of a response. All fixations made before this, including those that visit the target area but do not belong to the last visit (run) of the target area, we considered to be searching fixations. This approach allowed us to address a number of questions: Can we confirm for natural scenes the previous findings that contextual cueing affects searching fixations (as a representative of attentional guidance) but not responding fixations? Is attentional guidance the mechanism at work in multiple-target cueing as well, or do we find a reduced number or duration of responding fixations, indicating the facilitation of decision and response processes as suggested by Wang et al. (2020)? If contextual cueing differs between contexts that are paired with one vs. two possible target locations, does the difference lie in attentional guidance (represented by searching fixations) and/or response processes (represented by responding fixations)? Lastly, if we find similar stark differences in the contextual cueing effect between major and minor target locations for multiple-target cueing to those reported in the existing literature, can these be explained by a difference in attentional guidance and/or response processes?

As a third question, we wanted to know whether the contextual cueing effect for multiple locations is restricted to specific locations for which, in principle, direct associations can be built. Alternatively, is it possible that a contextual cueing effect is exhibited for a particular region (or, in our case, a hemifield)? In the field of location-probability cueing, the probability of target locations in a visual search task is manipulated by presenting targets more frequently, e.g., in one quadrant than in the other three quadrants (cf., Miller, 1988; Geng and Behrmann, 2002; Jiang et al., 2013). Consequently, participants find the target more quickly when it is presented in the implicitly cued region. The crucial difference from contextual cueing is that the uneven target distribution is not specific to a particular search display but is realized across all search displays presented in the experiment. Here, we asked whether observers can learn to prioritize a similar selection context-sensitively rather than through the general selection history (cf., Awh et al., 2012; Anderson et al., 2021). Whether this is possible was unclear. On the one hand, the mere repetition of a context could facilitate search or discrimination of objects within it. Indeed, Vadillo et al. (2021) observed a search benefit for repeated configurational contexts in which the target appeared at random positions. On the other hand, following the model by Brady and Chun (2007), we can expect a contextual cueing effect for multiple specific locations, but not for regions. According to the model, presenting a target at a specific position results not only in an association between the output node for this specific position and the closest input node activated by a distractor present at its position, but also in the construction of an association between the output node and input nodes further away; however, this association is modulated by the distance between input and output node. Thus, although association formation is subject to a spatial restriction, this is not absolute. However, the converse restriction is absolute. When a target is at a certain position, the output nodes coding for the surrounding positions do not develop any associations with the activated input nodes in the environment. Hence, it is an open question as to whether a contextual cueing effect for a cued hemifield can develop in natural scenes.

To sum up, the current study aimed to test whether pairing a natural scene context with more than one possible target location leads to effective contextual cueing. Specifically, we intended to investigate whether a positive contextual cueing effect would emerge for all possible targets or whether it would be reserved for a single target location, leading to costs for other possible target locations. At the same time, we wanted to clarify whether attentional guidance and/or response-related processes are affected by contextual cueing of one- and/or two-target locations.

To this end, we conducted two experiments. In the first experiment, we replicated the contextual cueing effect for one consistent target location in natural scenes, adapting Experiment 1a of the study by Brockmole and Henderson (2006b). Additionally, we applied the distinction between searching fixations (attentional guidance) and responding fixations (response-related processes) and found that the contextual effect was observed in the former but not the latter type of fixations. In Experiment 2, we could then compare the contextual cueing effect between a condition with one possible target location, a condition with two possible target locations, and a condition with many target locations restricted to one hemifield. This last condition was intended to explore the flexibility of contextual cueing in natural scene contexts even further. Can only specific locations be prioritized contingent on a concrete natural scene context, or can regions/screen side also be prioritized? During analysis, a specific focus was placed on the relationship between the two locations in the condition in which the target location alternated between two possible target locations. Our results show that, although contextual cueing arose somewhat later during the learning process, the effect became visible for both target locations, developing from an early negative to a positive effect even for the less-favored location after some training (approximately eight instances of possible learning). Distinguishing between searching and responding fixations allowed us to clarify that a reduced amount of contextual cueing was attributable to less efficient guiding (a smaller contextual cueing effect in cumulative searching fixations for the less favored target location) rather than to hindering of identification and initiation of the response for one-target-location as well as for multiple-target-location cueing. A further novel finding was that within the two-target-contexts, the different degrees of contextual cueing between major and minor target locations lay in less efficient guiding toward the minor location rather than a prolonged response-selection process, according to the data on searching and responding fixations.

## 2 Experiment 1

Experiment 1 was designed to replicate the results of Brockmole and Henderson (2006b), who showed that participants exhibited a substantial contextual cueing effect for search in real-world scenes that were related to arbitrary (i.e., independent of scene syntax or semantics) target locations compared to novel scenes in which no relation to specific target locations existed. In their Experiment 1b, they showed that this pronounced contextual cueing effect (>2 s) was accompanied by a high rate of recognition of repeated scenes, which differed from the recognition rate for once-seen and new scenes, as well as more accurate memory of the target location in repeated compared to once-seen scenes. In our study, eight scenes (real-world photographs) were each paired with one target location. These scenes, with this target location, were repeated 16 times across the experiment, constituting the repeating contexts. Another 128 scenes were shown exactly once with one target location, constituting the novel contexts.

Beyond the replication, we introduced high-resolution eye-tracking to this paradigm, allowing us to more closely examine the processing locus of contextual cueing. We based our eye-tracking analysis on two types of fixations: searching fixations (defined as all fixations that were made after the onset of the search display and before the first fixation of the last run on the target area of interest) and responding fixations (defined as all fixations that were made until the manual response, starting with the first fixation of the last run on the target area of interest). Should the repetition of the context affect searching fixations in number and/or duration, this would indicate that contextual cueing takes place in attentional guidance during the phase in which the observer is trying to find the target. In contrast or additionally, should responding fixations be affected by context repetition, this would indicate that processes related to response preparation for the target (also) play a role in the contextual cueing effect.

### 2.1 Methods

#### 2.1.1 Participants

We determined the appropriate sample size for Experiment 1 by starting from the size of the contextual cueing effect in Experiment 1 of the study by Brockmole and Henderson (2006b). The effect size of the main effect of trial type (repeated vs. novel) was $\eta_{\text{p}}^{2}$ = 0.24 corresponding to *f* = $\sqrt{\frac{\eta_{p}^{2}}{1\;-\eta_{p}^{2}}}$ = 0.562. We used this effect size to calculate a minimum sample size of 10 participants in our Experiment 1 comprising 2 conditions (assuming α = 0.05 and 1–β = 0.85, calculated with G^*^power; Faul et al., 2009). To guarantee a large enough sample, even in the potential case of missing data, we aimed to test 16 participants, a sample size comparable with those of other contextual cueing experiments (e.g., Zellin et al., 2011). Ultimately, 17 participants (3 male; median age: 22 years) volunteered for Experiment 1 in return for course credit or payment of 2.50 € per quarter hour. Participants had normal or corrected-to-normal visual acuity (glasses or contact lenses) and intact color vision. All but one were right-handed. They gave written informed consent before participation. The type of experiment was approved by the ethics committee of Bielefeld University.

#### 2.1.2 Apparatus and stimuli

Participants performed the experiment in a semi-lit room. They rested their head on a head- and chinrest to ensure that they viewed the computer screen from a distance of 71.8 cm (G90fB, ViewSonic, Brea, CA, USA). The screen had a resolution of 1,024 × 768 pixels at physical dimensions of 36 × 27 cm, corresponding to 26.88° × 20.82° visual angle, and a refresh rate of 100 Hz; it was controlled by a GeForce GTX 970 graphics card (driver version 344.48, NVIDIA, Santa Clara, CA, USA).

A video-based tower-mounted eye-tracker (EyeLink 1000, SR Research, Ottawa, Ontario, CA) recorded the behavior of each participant's right eye at a sampling rate of 1,000 Hz. A nine-point grid calibration of the eye-tracker was performed at the beginning of the experiment, before the recognition test, and whenever a participant did not achieve successful fixation on the central fixation stimulus at the beginning of a trial for the fifth time in a row. The median average error during validation was 0.37 (0.4°) visual angle and the median maximum error was 0.79 (0.7°) visual angle in Experiment 1 (Experiment 2). Fixation was considered unsuccessful if participants' gaze exceeded the limit of 2.013° radius around the center of the fixation stimulus during the enforced fixation interval.

The participants responded using a standard computer mouse (Logitech RX300). The mouse was placed centrally in front of the participants, and they pressed the left and right mouse buttons with their left and right index fingers, respectively. The experimental paradigm was programmed using the Psychophysics toolbox (3.0.12; Brainard, 1997; Pelli, 1997; Kleiner et al., 2007) and the Eyelink toolbox (3.0.12; Cornelissen et al., 2002) extensions for MATLAB (R2014b; The MathWorks, Natick, MA).

A black square (0.227° × 0.227°, L = 4.13 cd/m^2^) was used as the central fixation stimulus, shown against a gray background (averaged across left and right stimulus locations: L = 38 cd/m^2^). Real-world scene stimuli were 210 full-color photographs of real-world scenes taken from the validation images of the Places365-Standard set of the Places Database (Zhou et al., 2018). Images were pseudo-randomly drawn from the set and selected according to the following criteria: each was required to be a photograph that was taken from the approximate perspective of a human observer, with an angle such that it covered a section of the environment wide enough to be called a scene (such as an overview of a room, or a landscape); and no humans and no clear letters were visible in the photograph. Additionally, half of the photographs were indoor and half were outdoor scenes. The selected photographs were cropped and resized to a resolution of 1,024 × 768 pixels to be shown full-screen. Each search display consisted of a scene image with a target letter superimposed on it. Each target stimulus was a light or dark gray “T” or “L” presented at a size of 0.34° visual angle diameter. Depending on the mean luminance of the area of the photo over which the letter was superimposed, the light or dark gray version of the target letter was used. The target letter could be placed anywhere in the image, keeping a distance of at least 1° from the edge of the image. To prevent location-probability effects, in every condition, half of the target locations were in the left half of the screen and half in the right half. The median distance between all target locations used for one participant was 11.96° (11.93°) visual angle in Experiment 1 (Experiment 2). Additionally, scene selection was controlled such that for every condition, half of the scenes were indoor and half were outdoor scenes.

#### 2.1.3 Procedure and design

##### 2.1.3.1 Trial sequence

**Figure 1:**
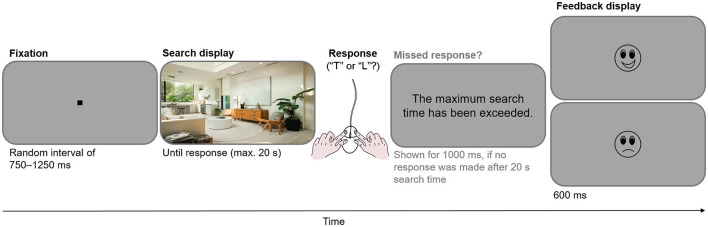
Sequence of a search task trial.

At the beginning of each trial, the participant viewed the central fixation stimulus for 250 ms; subsequently, they were required to fixate on this stimulus within a 2.013° radius for a random duration between 500 and 1,000 ms (which was a multiple of 10 ms). If the participant's gaze moved outside this radius, they were prompted to look at the center more accurately, and the trial was aborted and repeated at a random position within the stream of subsequent trials in the current block. Following the fixation stimulus, the search display was presented. The participant's task was to search the display for a “T” or “L” and to indicate the letter's identity as quickly as possible by clicking the right or left mouse button. The search display remained on the screen until a response was recorded, but at most until 20 seconds had elapsed since the search display onset. Participants received feedback as to whether they had answered correctly and on time. Figure 1 illustrates the trial sequence.

##### 2.1.3.2 Design

A two-factorial within-participant design was used with context and epoch as independent variables. Context had two levels, *repeated* and *novel*. For the *novel* contexts, 128 scene images were randomly selected from the pool of 216 photographs, and 128 target locations were randomly determined. For the *repeated* context condition, eight different scene images were selected. Each of the eight scenes, i.e., each context, was assigned one randomly determined location. The identity of the target letter (“T” or “L”) was determined randomly for each trial, whereas the combination of scene and target location remained the same in the *repeated* context condition. Under the second variable, epoch, the experimental trials were divided into four consecutive bins, to allow us to assess possible learning effects over the course of the experiment. Each epoch consisted of four blocks; in turn, each block contained eight *repeated* context trials (representing one repetition of a *repeated* context) and eight *novel* context trials. Within each block, trials from both conditions were randomly intermixed and the order of scenes was randomly generated for each block. There were 16 blocks in the experiment, with 256 trials in total. The mapping of target letters to response buttons was counterbalanced across participants.

##### 2.1.3.3 Recognition test

**Figure 2:**
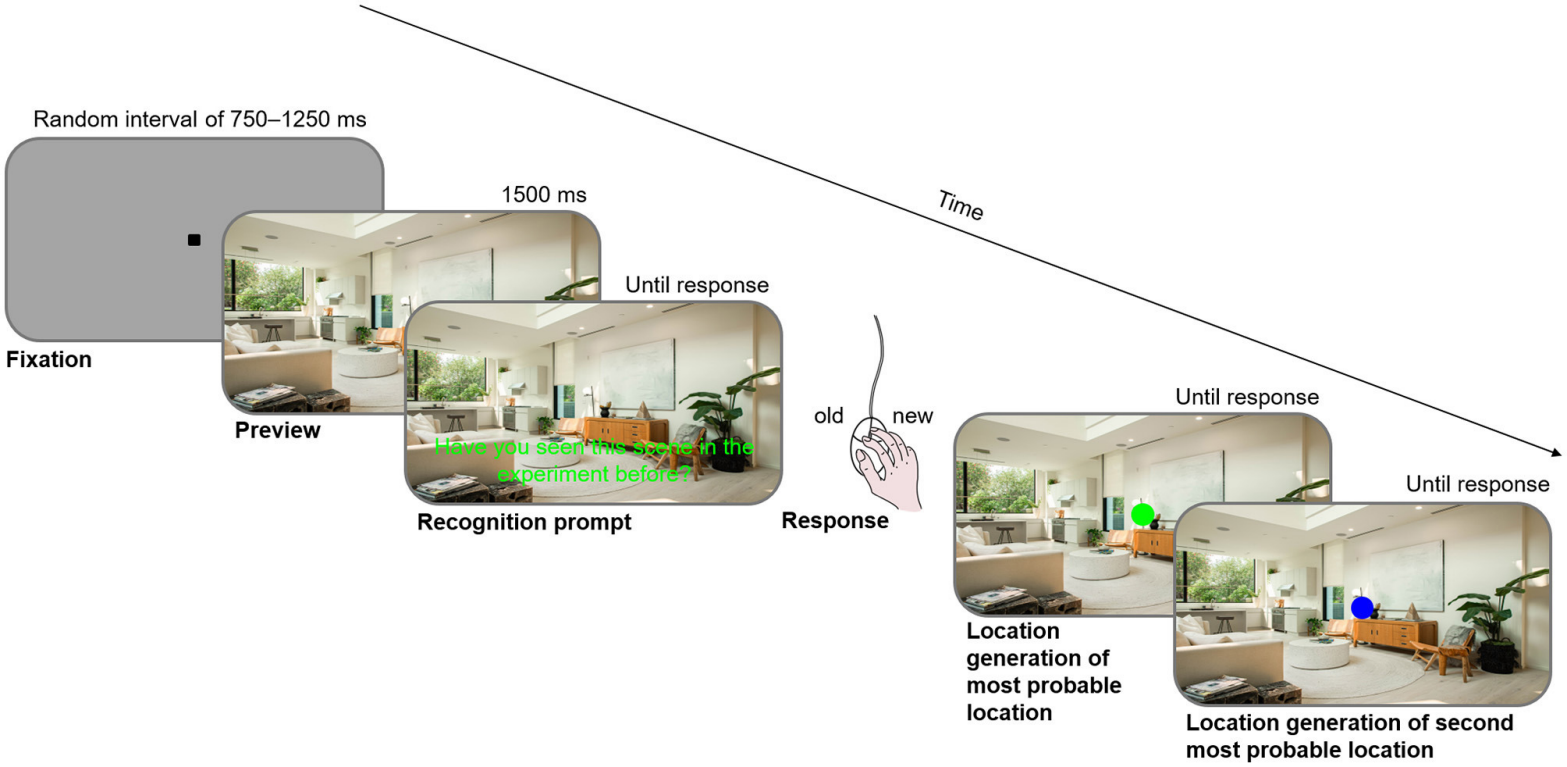
Sequence of a recognition test trial.

After completing the search task (after block 16), participants performed a recognition test corresponding to Experiment 1b by Brockmole and Henderson (2006b). In a randomized order, participants were presented with eight repeated contexts and sixteen novel scenes that had not been shown before. After establishing central fixation, a full-screen preview of one scene was presented for 800 ms; participants were then prompted to answer via mouse-click whether they had seen the scene in the experiment before. Subsequently, a green circle of approximately the size of the target letter appeared. Participants used the computer mouse to navigate the circle to the position where they would have expected the target letter to be, and confirmed this by clicking the left mouse button. They were asked to indicate a likely location even when they had not seen the scene before or they had no idea as to a likely location. Figure 2 illustrates the sequence of a recognition test trial for Experiment 2, which was identical to the sequence for Experiment 1 except that the last step was only present in Experiment 2.

#### 2.1.4 Data analysis

Pre-processing of raw gaze data was performed using the EyeLink Data Viewer (4.2.1). Further pre-processing and statistical analysis was performed using R (version, 4.1.2; R Core Team, 2021). Differences across conditions of two or more independent variables were examined by performing repeated-measures analyses of variance (ANOVAs) with type III sum of squares, followed by *post-hoc* paired-samples *t*-tests *(aggregated on the participant level)*. To prevent alpha error accumulation, a Bonferroni-corrected significance level was applied if multiple comparisons were performed. If the assumption of normal distribution was not met, the Wilcoxon signed-rank test was performed as the *post-hoc* procedure. The ANOVA assumption of sphericity was tested using Mauchly's test for sphericity. In cases of non-sphericial data, Greenhouse–Geisser correction was used and the applied correction factor ε is reported. As a measure of effect size, Cohen's *d*_z_ was calculated for *t*-tests (Cohen, 1988, p. 48), $\eta_{\text{G}}^{2}$ for ANOVAs (Bakeman, 2005), and *r* for Wilcoxon signed-rank tests (e.g., Field et al., 2012, p. 673). In addition to conventional *t*-tests, the BayesFactor package (0.9.12-4.2; Morey et al., 2018) for R (version, 4.1.2; R Core Team, 2021), cf. Rouder et al. (2009), was used to compute Bayes factors (*BF*_10_) using current standard settings for paired designs as a further *post-hoc* procedure. To increase statistical power, data were aggregated into four epochs, each containing four consecutive blocks.

#### 2.1.5 Eye-movement analysis

Saccades were detected by a combined velocity (35° × s^−1^) and acceleration (9,500° × s^−2^) threshold. A fixation was defined as any period that was not a saccade or a blink (a period in which pupil data were missing for three or more consecutive samples). No minimum distance for saccades or minimum duration for fixations was set.

A saccade was considered as landing on the target if it was within a radius of 2.0° around the target's center. Using this target area of interest, the start time of the last run on the target, i.e., the last time the target area of interest was entered (and potentially left), was determined. The first fixation of the last run was considered the first of the *responding fixations*. The last responding fixation was considered the last fixation in the trial before a response was made. Correspondingly, the *searching fixations* were defined as all fixations made from the onset of the search display until (but excluding) the first fixation of the last run on the target. Concerning fixation durations, we determined for every participant for every trial the median durations of *responding fixations* and *searching fixations*.

### 2.2 Results

#### 2.2.1 Repeated trials

The total proportion of trials that were repeated because of erroneous fixation of the initial fixation stimulus was 12.43% in the search task and 12.07% in the recognition test.

#### 2.2.2 Trial exclusion and missing data

Pooled over all participants, there were 4,352 trials in the search task. In 294 trials, no answer was given; these trials were excluded from analysis. An additional 137 trials were excluded because the participant gave an incorrect response. Based on the remaining trials, each participant's mean manual response time and the corresponding standard deviation were calculated, and 102 trials in which the manual response time deviated from the mean by more than 3 standard deviations were excluded. Manual response time analysis was thus performed on 3,819 trials.

For the analysis of eye-movement data, among the remaining trials, the number and duration of searching and responding fixations could not be determined in 70 cases, because no fixation within the target interest area was registered. In another 57 trials, the first fixation was also the first fixation of the last run on the target area of interest, so there were no searching fixation durations to be measured. Thus, analysis of the number of searching and responding fixations (including the cases in which there were zero searching fixations) was performed on 3,749 trials. Analysis of fixation duration was performed on 3,749 trials in the case of responding fixations and on 3,692 trials in the case of searching fixations.

#### 2.2.3 Response accuracy

On average, participants failed to provide an answer before 20 s had passed in 6.76% of the trials (SD = 4.79%). Among the remaining trials, participants gave accurate answers (e.g., “T” when the target was a “T”) on average in 96.57% of the trials (SD = 1.92). Neither the proportion of misses nor accuracy differed between conditions (for details, see Section 1.1.1 of the Supplementary material).

#### 2.2.4 Manual response time

##### 2.2.4.1 Equality check in first block

Since the context conditions only differed in regard to whether scenes were repeated, manual response time was not expected to differ between context conditions during the first block, in which each scene was presented for the very first time regardless of context condition. A Bayesian *t*-test confirmed that manual response time did not differ between scenes consisting of *repeated* or *novel* contexts in block 1, *BF*_10_ = 0.288, *d*_z_ = 0.112.

##### 2.2.4.2 Effects of context and epoch

**Figure 3:**
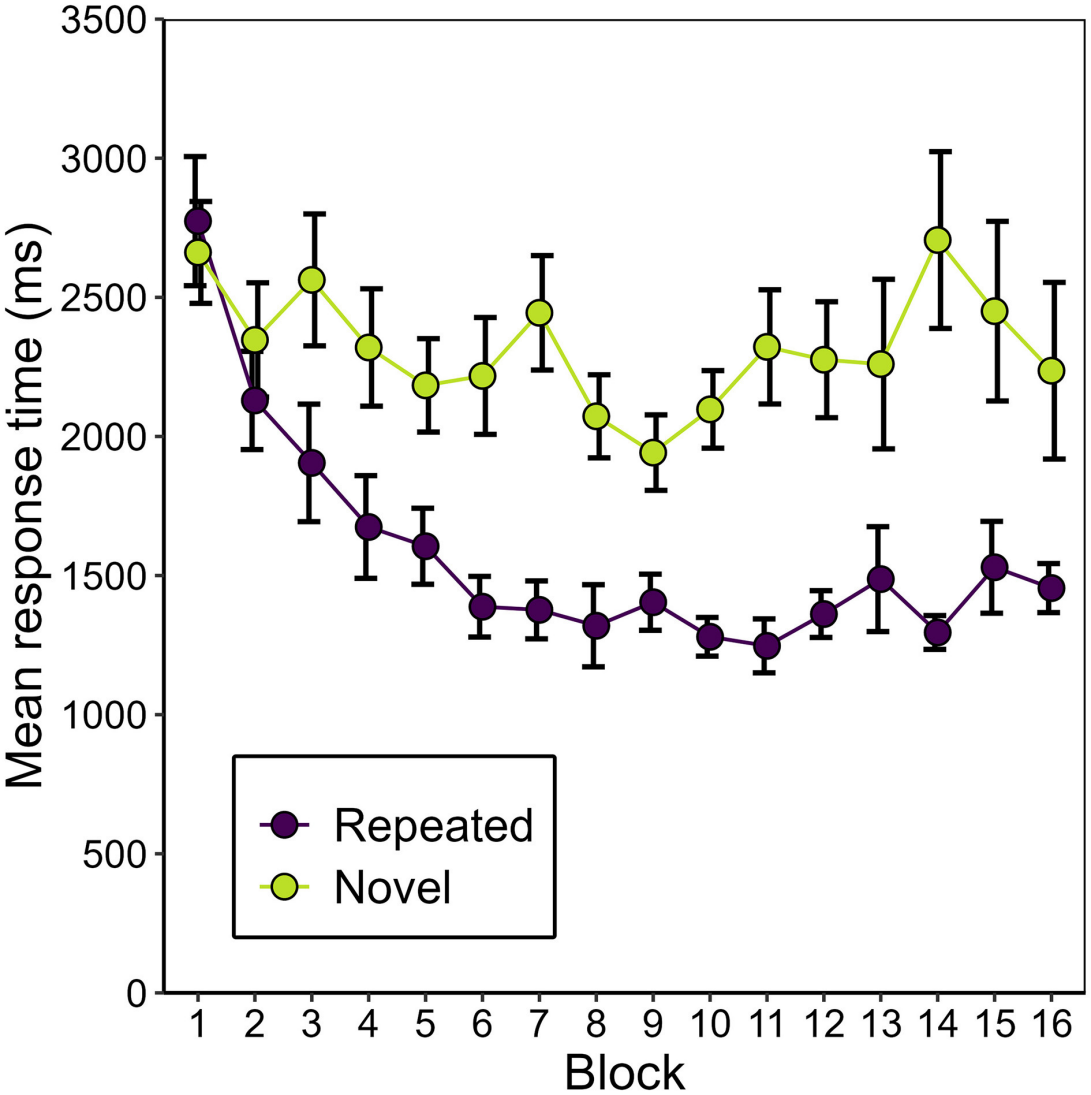
Mean manual response time (mean of the participants' average search time across scene identities) as a function of context (*repeated* or *novel*) and block in Experiment 1. Error bars represent standard errors for within-subjects designs (Morey, 2008).

**Figure 4:**
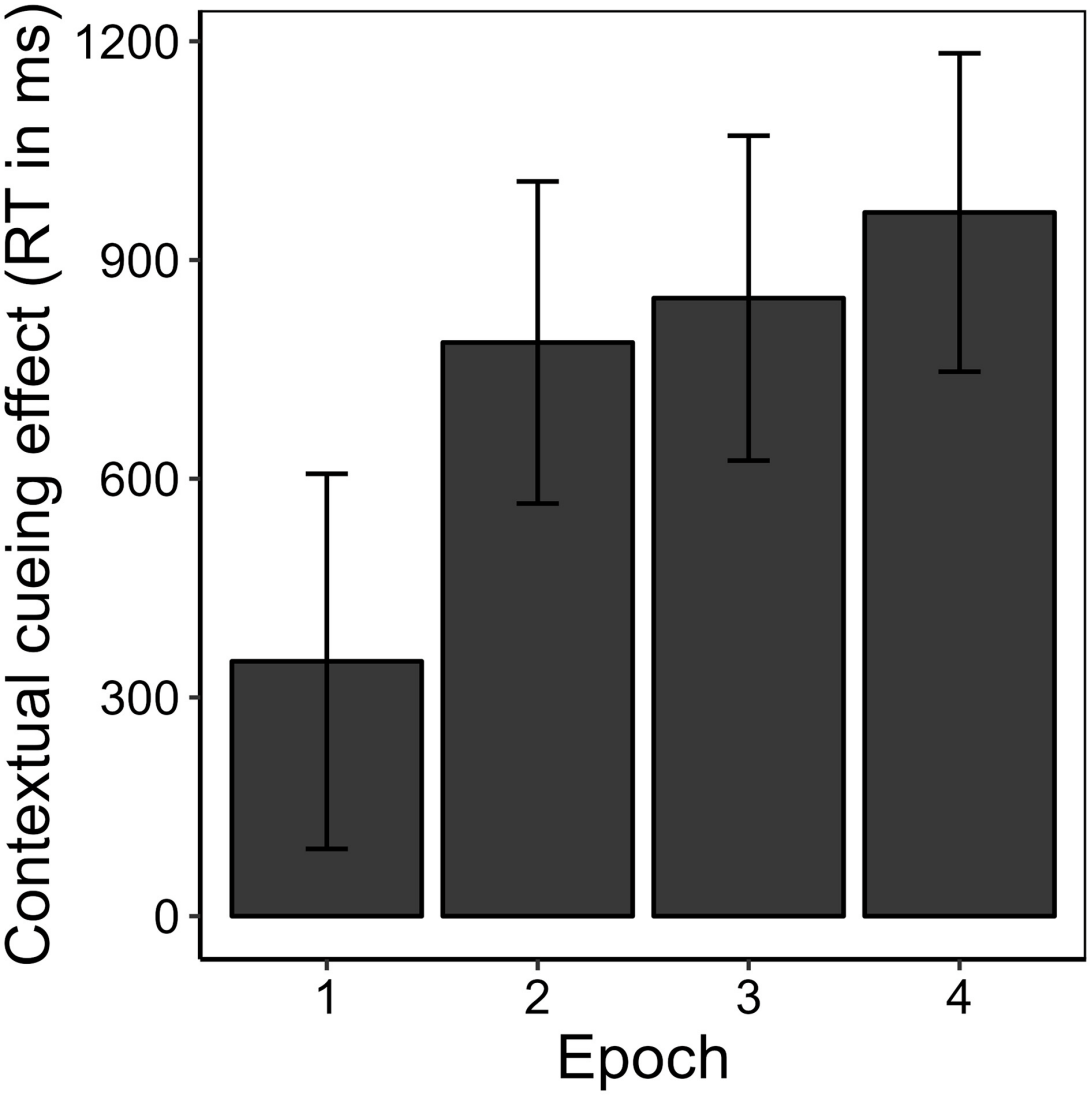
Contextual cueing effect as the mean difference in mean manual response times (RT) in *novel* and *repeated* contexts for each epoch in Experiment 1. Error bars represent 95% confidence intervals for within-subjects designs (Morey, 2008).

Figure 3 shows the development of manual response time over the course of the experiment, separately for *repeated* and *novel* contexts. Correcting for multiple comparisons made on the manual response time data, we applied a Bonferroni-corrected alpha level of α_corr_ = 0.0056. A 2 (*repeated* vs. *novel*) × 4 (epoch) repeated-measures ANOVA revealed a main effect of context, *F*_(1, 16)_ = 33.71, *p* < 0.001, $\eta_{\text{G}}^{2}$ = 0.154. The effect of epoch missed significance, *F*_(1.28, 20.56)_ = 3.69, *p* = 0.060, ε_GG_ = 0.428, $\eta_{\text{G}}^{2}$ = 0.056. However, context and epoch interacted significantly, *F*_(2.30, 36.86)_ = 6.12, *p* = 0.004, ε_GG_ = 0.768, $\eta_{\text{G}}^{2}$ = 0.018. Two separate *post-hoc*, one-way repeated-measures ANOVAs showed that this interaction was grounded in a significant effect of epoch for *repeated* contexts, *F*_(1.29, 20.58)_ = 12.01, *p* = 0.001, ε_GG_ = 0.429, $\eta_{\text{G}}^{2}$ = 0.206, but not for *novel* contexts, *F*_(1.50, 23.96)_ = 0.79, *p* = 0.433, ε_GG_ = 0.499, $\eta_{\text{G}}^{2}$ = 0.014. *Post-hoc* pair-wise comparisons showed that manual response times were significantly faster in *repeated* than in *novel* contexts in epochs 2 to 4 [epoch 2: *t*_(16)_ = −4.64, *p* = < 0.001, *BF*_10_ = 117.76, *d*_z_ = −1.13; epoch 3: *t*_(16)_ = −6.73, *p* < 0.001, *BF*_10_ = 4288.02, *d*_z_ = −1.63; epoch 4: *t*_(16)_ = −5.28, *p* < 0.001, *BF*_10_ = 368.78, *d*_z_ = −1.28], but that this was not yet the case in epoch 1 [*t*_(16)_ = −2.36, *p* = 0.032, *BF*_10_ = 2.12, *d*_z_ = −0.57]. This finding represents a positive contextual cueing effect that developed over time (see Figure 4). On average, manual response times were 737 ms (*SD* = 678 ms) shorter in *repeated* contexts than in *novel* contexts (*M* = 2,315 ms, *SD* = 1,068 ms).

#### 2.2.5 Searching and responding fixations

##### 2.2.5.1 Number of fixations

**Figure 5:**
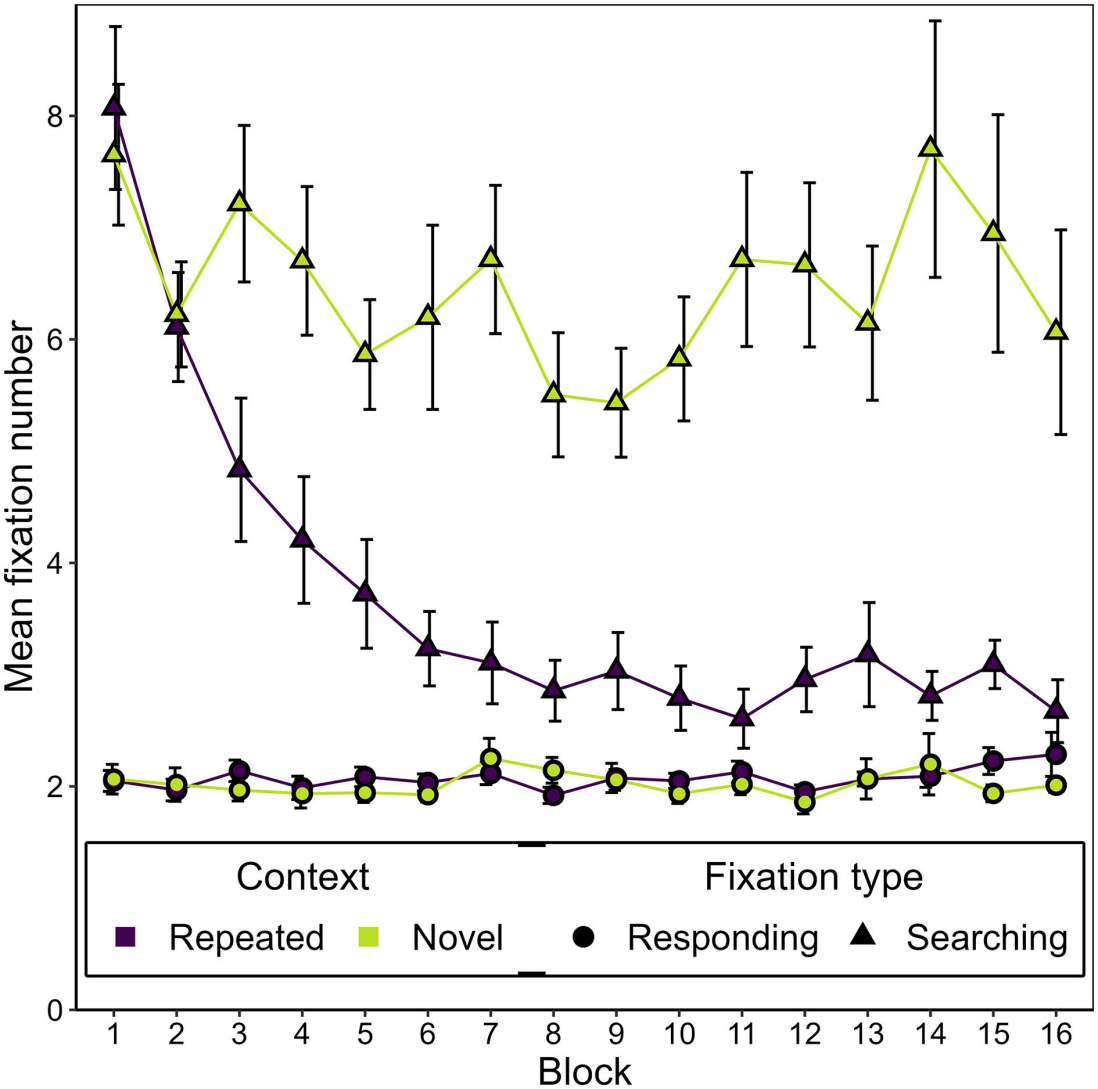
Mean number of responding and searching fixations as a function of block and context in Experiment 1. Error bars represent standard errors for within-subjects designs, calculated considering each fixation type separately (Morey, 2008).

Figure 5 shows the development of the mean number of responding and searching fixations over the course of the experiment, separately for *repeated* and *novel* contexts. Two observations leap to the eye. First, we observed a generally lower number of responding fixations (*M* = 2.05, *SD* = 0.48) than searching fixations (*M* = 5.08, *SD* = 2.54), *t*_(16)_ = −14.24, *p* < 0.001, *BF*_10_ = 53,336,987.00, *d*_z_ = −3.45.

Second, we noticed an interaction of block and context affecting the number of searching fixations, with searching fixations decreasing over time for *repeated* but not *novel* contexts. A two-way repeated measures ANOVA on the number of searching fixations confirmed this interaction effect, *F*_(2.32, 37.17)_ = 7.89, *p* < 0.001, ε_GG_ = 0.774, $\eta_{\text{G}}^{2}$ = 0.061, alongside significant main effects of context, *F*_(1, 16)_ = 40.01, *p* < 0.001, $\eta_{\text{G}}^{2}$ = 0.34, and epoch, *F*_(1.82, 29.19)_ = 7.43, *p* = 0.003, ε_*GG*_ = 0.608, $\eta_{\text{G}}^{2}$ = 0.13. In contrast, for the number of responding fixations there was neither a significant effect of context, *F*_(1, 16)_ = 0.93, *p* = 0.349$,\eta_{\text{G}}^{2}$ = 0.004, nor an effect of epoch, *F*_(1.26, 20.21)_ = 0.398, *p* = 0.583, $\eta_{\text{G}}^{2}$ = 0.006; nor was there an effect of their interaction, *F*(2.75, 44.03) = 1.10, *p* = 0.357, $\eta_{\text{G}}^{2}$ = 0.004. *Post-hoc* one-way ANOVAs showed that the number of fixations significantly changed over epochs only in the case of the searching fixations and only in the *repeated* contexts, but not in *novel* contexts and not for responding fixations (see Table 1). As can readily be seen in Table 1, this pattern of effects is due to a decline in the number of searching fixations in repeated contexts over the course of the experiment, resulting in significant contextual cueing effects on the number of searching fixations in epoch 2, *t*_(16)_ = −5.55, *p* < 0.001, *BF*_10_ = 590.25, *d*_z_ = −1.35, epoch 3, *t*_(16)_ = −6.55, *p* = < 0.001, *BF*_10_ = 3,181.12, *d*_z_ = −1.59, and epoch 4, *t*_(16)_ = −5.48, *p* = < 0.001, *BF*_10_ = 519.42, *d*_z_ = −1.33. For epoch 1, no significant contextual cueing effect could be observed, *t*_(16)_ = −2.28, *p* = 0.036, *BF*_10_ = 1.88, *d*_z_ = −0.55. For the number of responding fixations, the same comparisons yielded evidence that no contextual cueing effect was present in any epoch (epoch 1: *BF*_10_ = 0.40, epoch 2: *BF*_10_ = 0.26, epoch 3: *BF*_10_ = 0.38, epoch 4: *BF*_10_ = 0.56).

**Table 1:** *Post-hoc* one-way ANOVAs with epoch as predictor of number of fixations.

**Group**								
**Fixation type**	**Context**	**Predictor**	**df**_Num_	**df**_Den_	ε_GG_	***F***	***p***	$\eta_{\text{G}}^{2}$
Searching	Novel	Epoch	3	48	0.021	0.814	0.492	0.019
	Repeated	Epoch	2.04	32.62	0.586	34.596	< 0.001	0.284
Responding	Novel	Epoch	1.68	26.83	0.008	0.418	0.627	0.017
	Repeated	Epoch	1.49	23.89	0.011	0.621	0.501	0.034

*df*_Num_ indicates numerator degrees of freedom. *df*_Den_ indicates denominator degrees of freedom. ε_GG_ indicates the Greenhouse–Geisser multiplier for the degrees of freedom; *p*-values and degrees of freedom in the table incorporate this correction. $\eta_{\text{G}}^{2}$ indicates generalized eta-squared.

##### 2.2.5.2 Fixation durations

**Figure 6:**
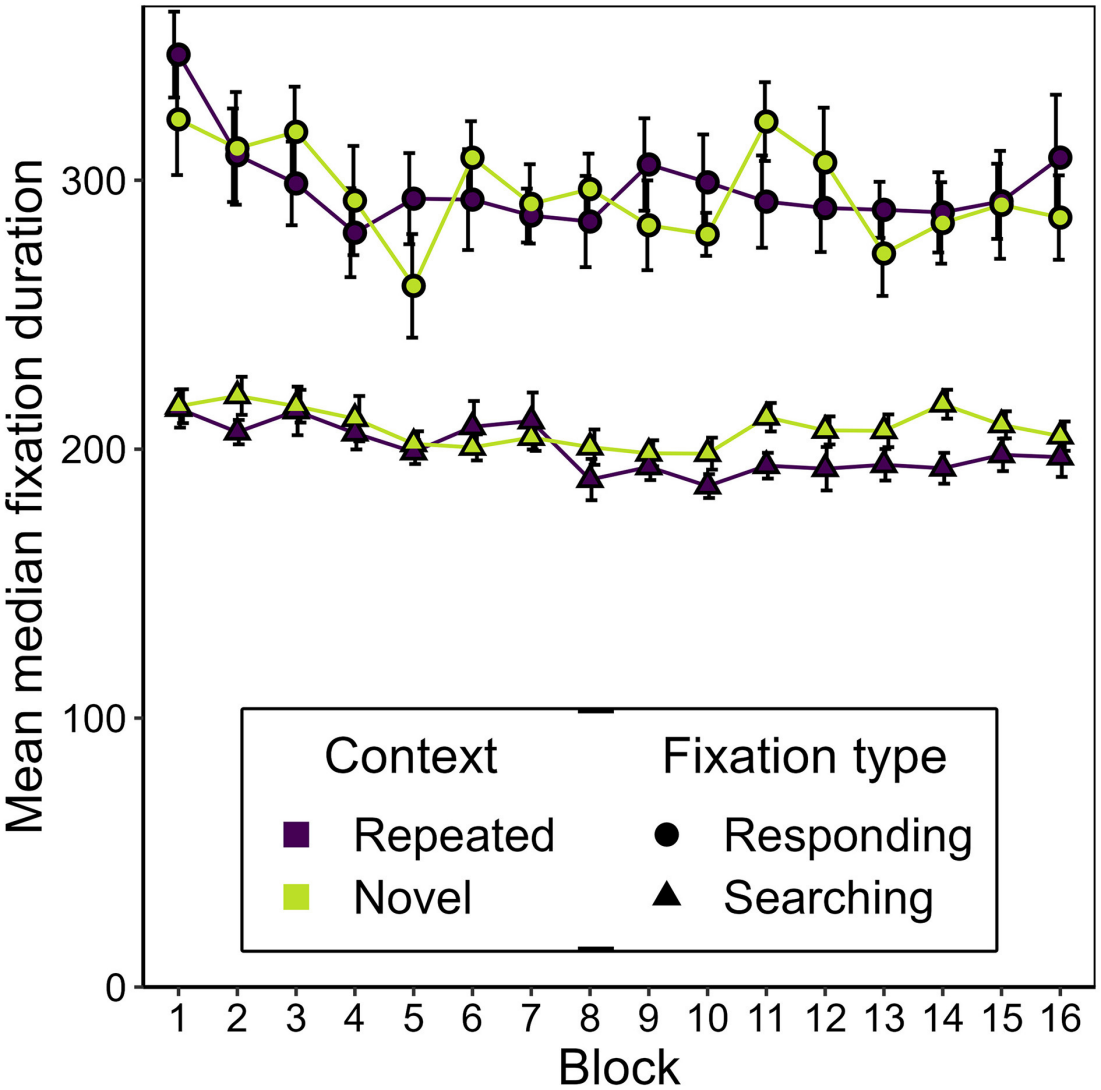
Mean median duration of *searching* and *responding* fixations as a function of block and context in Experiment 1. Error bars represent standard errors for within-subjects designs, calculated considering each fixation type separately (Morey, 2008).

Figure 6 shows the development of the mean median duration of responding and searching fixations over the course of the experiment, seperately for *repeated* and *novel* contexts. Overall, the two fixation types differed significantly, as fixation duration was generally lower for searching fixations (*M* = 204 ms, *SD* = 35 ms) than for responding fixations (*M* = 296 ms, *SD* = 64 ms), *t*_(16)_ = −6.75, *p* = < 0.001, *BF*_10_ = 4,372.17, *d*_z_ = −1.64.

We expected the durations of *responding* fixations to decrease early in the experiment as a consequence of discrimination practice. However, we did not observe a significant effect of epoch on responding fixation durations, *F*_(1.26, 20.21)_ = 0.40, ε_GG_ = 0.421, *p* = 0.583. Nor was a difference observed in responding fixation durations between *novel* and *repeated* contexts, *F*_(1, 16)_ = 0.93, *p* = 0.349, *BF*_10_ = 0.14.

For searching fixations, however, context showed a trend indicating an effect on duration, *F*_(1, 16)_ = 5.46, *p* = 0.033, $\eta_{\text{G}}^{2}$ = 0.13, and there was a significant effect of epoch, *F*_(2.69, 43.02)_ = 5.73, ε_GG_ = 0.896, *p* = 0.003, $\eta_{\text{G}}^{2}$ = 0.022, but no interaction, *F*_(1.91, 30.58)_ = 5.73, ε_GG_ = 0.896, *p* = 0.307. Evidence for a difference in fixation durations between contexts slightly outweighed the alternative, *BF*_10_ = 4.34. Further Bayesian *t-*tests showed that this difference was only visible in epoch 4, *BF*_10_ = 8.57, and not earlier (epoch 1: *BF*_10_ = 0.37, epoch 2: *BF*_10_ = 0.25, epoch 3: *BF*_10_ = 1.39). This might represent a hint of a contextual cueing effect on searching fixation duration that develops over time.

##### 2.2.5.3 Cumulative fixation durations

**Figure 7:**
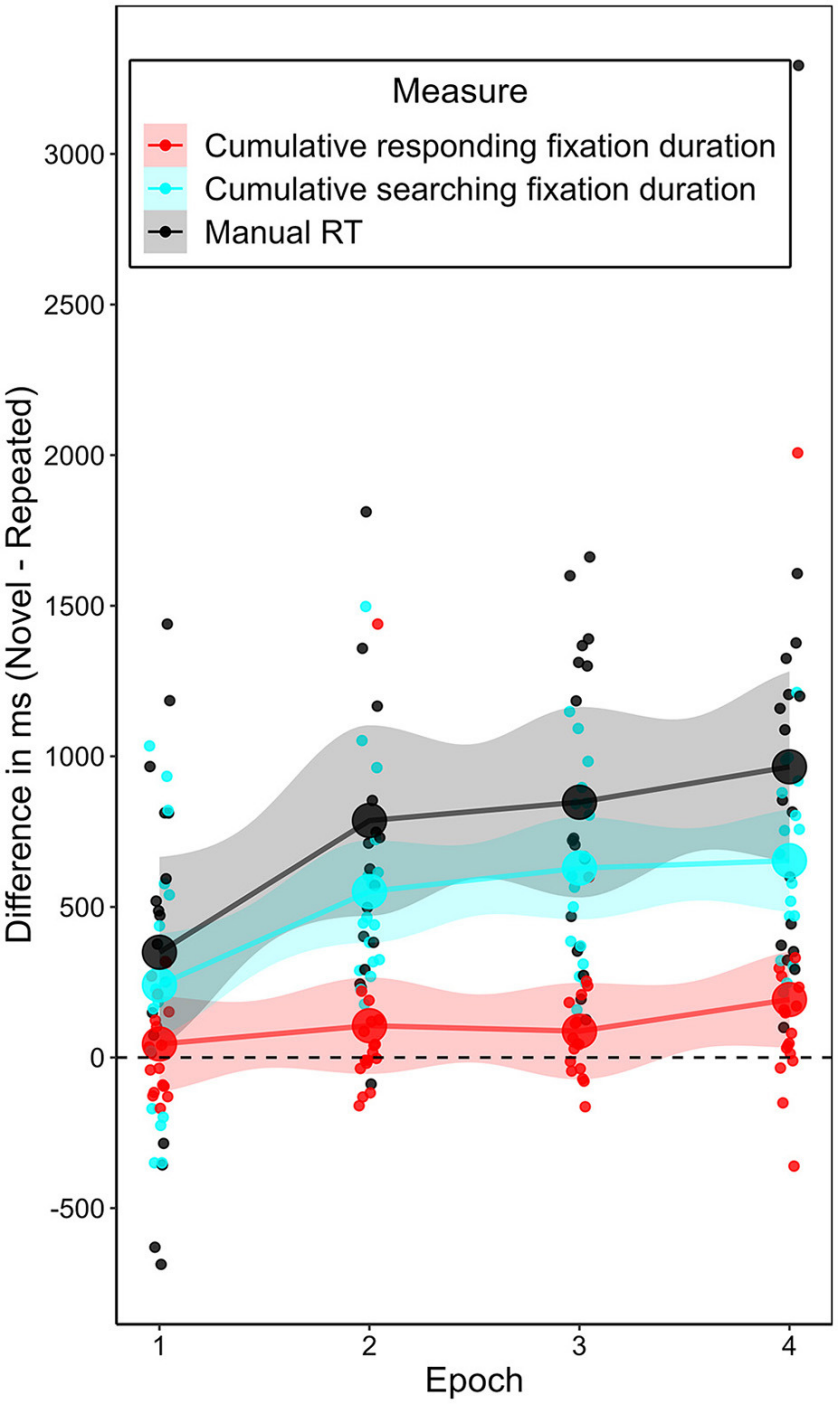
Development of the contextual cueing effect on manual response time and the two fixation types. Depicted is the mean difference in mean duration (manual response time, cumulative searching fixations, cumulative responding fixations) between *novel* and *repeated* contexts per epoch in Experiment 1.

Having observed a significant contextual cueing effect on manual response time and on the number of searching fixations, but not on the number of responding fixations, we wanted to explore the relationships between the contextual cueing effect on response time and the effects on the different fixation types. To establish three comparable measures, we considered the cumulative duration of searching and responding fixations, respectively. Figure 7 puts these and manual response time in context. The analyses so far suggested that the contextual cueing effect on response time develops in parallel with a contextual cueing effect on searching fixations, while being relatively independent of the development of an effect on responding fixations. To corroborate the assumption of a connection between the contextual cueing effect on response time and the effect on searching fixations, three observations would be necessary: (1) the sum of the cumulative searching and responding fixation durations should be smaller than or equal to the manual response time in all trials; (2) the cumulative searching fixation duration should be lower in *repeated* contexts than in *novel* contexts; and (3) this difference should increase over the course of the experiment. With respect to (1), the summed cumulative fixation durations were always lower than or equal to the manual response time. With respect to (2), as for the number of fixations, the cumulative duration of searching fixations was also significantly lower in the *repeated* contexts than in the *novel* contexts, *t*_(16)_ = 8.424, *p* < 0.001, *BF*_10_ = 56,300.58, *d*_z_ = 2.043. For completeness, this was not the case for the cumulative duration of responding fixations, *t*_(16)_ = 1.952, *p* = 0.069, *BF*_10_ = 1.153, *d*_z_ = 0.473. With respect to (3), as an example analysis of the development of the contextual cueing effect on the two fixation types, we analyzed the difference in the contextual cueing effect on each measure between epoch 1 and 2. Although restricting the analyses to epochs 1 and 2 inflated the alpha error, we chose this specific comparison to increase statistical power and because the largest learning effect in contextual cueing occurred between these two epochs. As such, between epoch 1 and 2, the largest increase was in contextual cueing effect on manual response time, increasing on average by 437 ms; *SD* = 706 ms; *t*_(16)_ = 2.515, *p* = 0.023, *BF*_10_ = 2.725, *d*_z_ = 0.610, see Figures 3, 7. For the searching fixations, the contextual cueing effect on their cumulative duration increased on average by 310 ms, *SD* = 515 ms, *t*_(16)_ = 2.449, *p* = 0.026, *BF*_10_ = 2.433, *d*_z_ = 0.593. For the responding fixations, the contextual cueing effect on their cumulative duration increased by 61 ms (*SD* = 414 ms), but the increase was not significant, *t*_(16)_ = 0.596, *p* = 0.559, *BF*_10_ = 0.291, *d*_z_ = 0.145. Comparing the effect sizes, it becomes obvious that while the contextual cueing effect on cumulative responding fixation duration showed a small, non-significant increase from epoch 1 to epoch 2, the size of the increase in the contextual effect on cumulative searching fixation duration was comparable to the increase in this effect on manual response time (see Figure 7).

To better illustrate the correspondence, we calculated the correlation between participants' mean contextual cueing effect on manual response time per epoch and their mean contextual cueing effect on cumulative searching or responding fixation duration per epoch (see **Table 3**). While the mean correlations were higher for searching (95% CI [0.67, 0.98]) than responding fixations (95% CI [0.20, 0.81]), the correlations did not differ significantly.

#### 2.2.6 Recognition performance

##### 2.2.6.1 Scene recognition

On average, participants reported remembering 99.27% of the repeated scenes (i.e., hits). Of the novel contexts, 2.21% were remembered (i.e., false alarms). Since a normal distribution could not be assumed, a Wilcoxon signed-rank test was performed. The percentage of the old contexts correctly recognized as old (*Mdn* = 100%) was significantly higher than the false alarm rate, i.e., the percentage of novel contexts recognized as old (*Mdn* = 0%; *z* = −3.745, *p* < 0.001, *r* = −0.642).

##### 2.2.6.2 Accuracy of location generation

**Figure 8:**
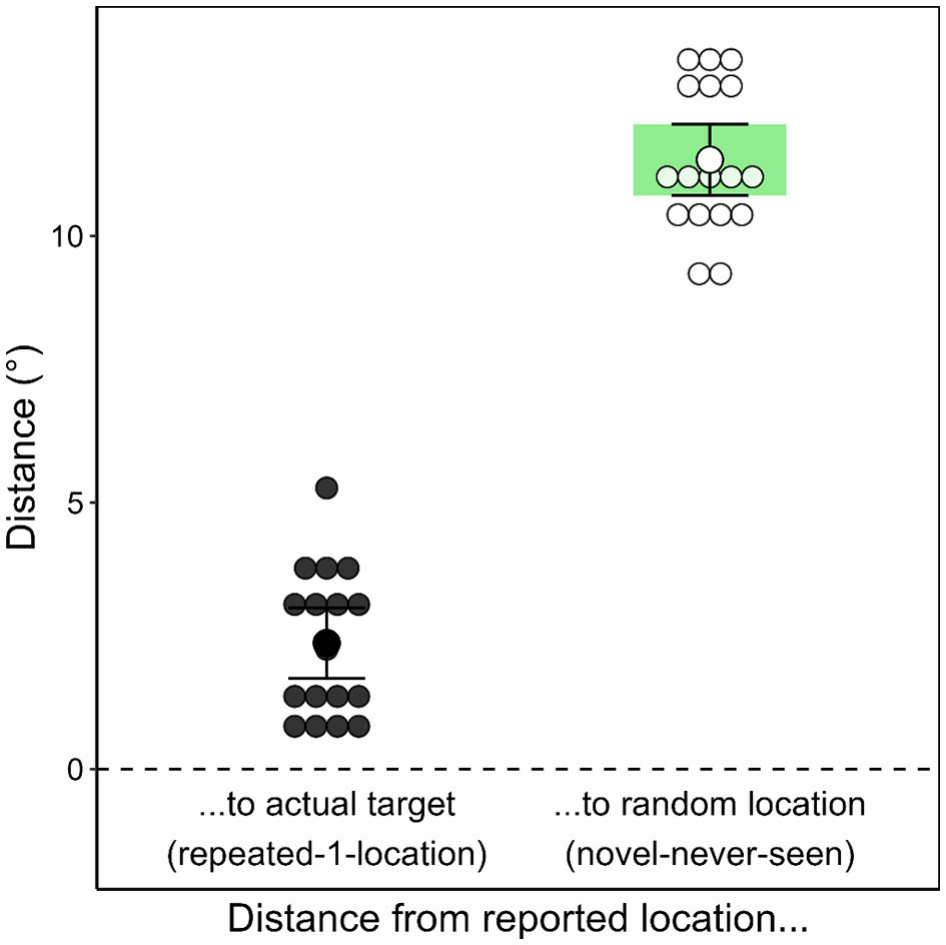
Accuracy of participants' target location generation in Experiment 1. The graph depicts the mean placement error by scene repetition for each participant, calculated as the distance in degrees of the visual angle between the generated location and the actual target location (when the scene was repeated) or a random dummy target location (when the scene had never been presented before).

The accuracy of the participants' memory for the target position (see Figure 8) was operationalized as the distance between the location generated in the recognition test and the actual location of the target during the search task or, for novel contexts, a dummy location (randomly generated location in the area of possible target locations). The placement error was significantly smaller for repeated contexts (*M* = 2.36 °, *SD* = 1.40°) than for novel contexts; *M* = 11.43°, *SD* = 1.39°; *t*_(16)_ = −18.003, *p* < 0.001, *d*_z_ = −4.366, *BF*_10_ = 1.44 × 10^9^.

### 2.3 Discussion

The results of Experiment 1 showed that contextual cueing in natural scenes (Brockmole and Henderson, 2006b) could be successfully replicated, including a much larger size for the cueing effect than observed in array-based search tasks (cf., Chun and Jiang, 1998; Jiang and Sisk, 2020). Beyond the replication, our eye-movement analysis revealed that context profoundly affected the number of searching fixations, but not the number of responding fixations. This provides convincing evidence that contextual cueing in natural scenes is largely localized in the early stage of search, i.e., the first phase, which should represent the attentional guidance process. The results regarding the duration of searching and responding fixations showed, if any, a small contextual cueing effect for searching fixations, but again no effect for responding fixations. More data are needed to examine whether context repetition indeed goes along with shortened searching fixation durations.

Having established that, in this variant of the contextual cueing paradigm, repetition of natural scenes elicits a large attentional guidance-based contextual cueing effect on searching for a target found at one repeated location, we examined in Experiment 2 whether this effect would extend to more than one repeated location—our second key question. Furthermore, it remained to be shown whether the novel finding would prove to be robust in terms of contextual cueing mainly affecting the number of searching fixations and not responding fixations, and to be clarified whether context repetition would also affect searching fixation duration if more blocks of repetition were added to the experiment.

## 3 Experiment 2

Experiment 1 confirmed that when targets are repeatedly found in a consistent, yet semantically and syntactically arbitrary position in a natural context, operationalized in the form of a photograph of a real-world scene, people find the target more quickly than in the case of novel scenes. This effect was again much more pronounced than the contextual cueing effect in arbitrary configurations (cf., Brockmole and Henderson, 2006b), and the results on fixation types suggested that the contextual cueing effect is routed in attentional guidance.

Experiment 2 investigated whether this attentional benefit extends to multiple locations in a given context and whether the selective effect of context repetition on searching but not responding fixations could also be confirmed in this case. From everyday life observation, we have established the notion that there are several possible object locations that are especially relevant in a given context, and that we efficiently orient ourselves to any of these locations, depending on the circumstances. However, studies based on arbitrary configurations have found smaller contextual cueing effects for multiple locations (e.g., Chun and Jiang, 1998; Kunar et al., 2005), or even costs for the less favored location (Zellin et al., 2011). Do these findings generalize to natural scenes, environments for which the visual system might be specialized to flexibly perform such a task?

In Experiment 2, we therefore asked whether in natural scenes there would be a contextual cueing effect for multiple locations in which the target is repeatedly and alternately found. To this end, we added a condition in which targets appeared in repeated scenes in two possible locations, alternating between these two locations with every block. In addition to this, we added a condition in which contexts were shown repeatedly but with the target always at a randomly generated location, restricted to either the left or the right hemifield. In this way, the condition was designed to tell us how far the flexibility of the cognitive system extends: i.e., whether it allows cueing not only of one or multiple specific locations but also of regions, contingent on the current context. Note that this might at first glance resemble location-probability cueing (cf., Miller, 1988; Geng and Behrmann, 2002; Jiang et al., 2013). However, spatial selection history was not manipulated in this condition of our experiment, since all target locations were uniformly distributed across all screen coordinates over the course of the experiment. Instead, it could in principle be beneficial to select the left or the right hemifield with higher priority, but this benefit was only contingent on the presence of a specific context.

### 3.1 Methods

#### 3.1.1 Participants

We determined the appropriate sample size for Experiment 2 by starting from the size of the contextual cueing effect in the *repeated-2-locations* condition in Experiment 2 in the study by Wang et al. (2020). The size of the main effect of context (*repeated* vs. *novel*) was $\eta_{\text{p}}^{2}$ = 0.30, corresponding to *f* = $\sqrt{\frac{\eta_{p}^{2}}{1\;-\eta_{p}^{2}}}$ = 0.655. We used this effect size to calculate a minimum required sample size of 6 participants in our Experiment 2, which comprised four conditions (assuming α = 0.05 and 1 – β = 0.85, calculated with G^*^power; Faul et al., 2009). In order to be able to observe—if present—an effect of context repetition in the condition in which we paired repeated contexts with new locations restricted to one hemifield, we adapted the sample size based on the estimated size of this effect. Based on the size of the contextual cueing effect for repeated displays associated with random locations in Experiment 2 of the study by Vadillo et al. (2021), namely, $\eta_{\text{p}}^{2}$ = 0.24, corresponding to *f* = 0.562, we calculated a minimum sample size of 12 participants (assuming α = 0.05 and 1 – β = 0.85, calculated with G^*^power; Faul et al., 2009).

Thus, comparable to Experiment 1, we aimed to test 16 participants. Eighteen different participants voluntarily participated in Experiment 2 for course credit or payment of 2.50 € per quarter hour. Data from one participant had to be discarded because of a software error, and data from another participant had to be discarded because they discontinued the experiment prematurely. Hence, a total of 16 participants (4 male; median age: 22.5 years) made up the sample. All had normal or corrected-to-normal visual acuity (glasses or contact lenses) and intact color vision. All were right-handed. They gave written informed consent before participation. The type of experiment was approved by the ethics committee of Bielefeld University.

#### 3.1.2 Apparatus and stimuli

The apparatus and stimuli were the same as in Experiment 1.

#### 3.1.3 Procedure and design

##### 3.1.3.1 Trial sequence

The trial sequence was the same as in Experiment 1.

##### 3.1.3.2 Design

In Experiment 2, a two-factorial within-participant design with context and epoch as independent variables was again used. However, context now had four levels: repeated with one location (*repeated-1-location)*, repeated with two locations (*repeated-2-locations*), repeated with locations in one hemifield (*repeated-1-hemifield*), and *novel*. For the *repeated-1-location* context condition, six scene images were randomly selected from the pool of 210 photographs. For the *repeated-2-locations* context condition, six different scene images were randomly selected, and each of the six scenes was assigned two randomly determined locations. Alternating with every block, the scenes were presented with the target in the one location or the other. That is, there were two sets of target locations for this condition, one used in uneven-numbered and one in even-numbered blocks. For the *repeated-1-hemifield* context condition, another six scene images were selected, and 180 (6 scenes × 30 blocks) locations were randomly selected. Each scene was repeated 30 times across the experiment, each time in combination with a different location. These locations were only restricted to all lie in the same hemifield (for 3 scenes in the right hemifield, and for 3 scenes in the left). The identity of the target letter was determined randomly for each trial. Under the second variable, epoch, the experimental trials were divided into six consecutive bins, to allow us to assess possible learning effects over the course of the experiment. Each epoch consisted of five blocks; each block contained a total of 24 trials, 6 trials for each context condition. In this way, one block represented one repetition of a scene; for the *repeated-1-location* contexts, it also represented one repetition of the pairing of a scene and a target location. For the *repeated-2-locations* contexts, two blocks represented a pairing of a scene with each of its assigned locations. Within each block, trials were drawn in random order from the four context conditions; within the context conditions, the order of the scenes was also randomly determined. There were 30 blocks in the search task of this experiment, with 720 trials in total. After each block, participants received progress information.

##### 3.1.3.3 Recognition test

The procedure for the recognition test was very similar to that of Experiment 1. The difference was that in Experiment 2, after participants had navigated the green circle to the position where they would have expected the target object most likely to be and clicked to confirm, the circle turned blue and participants navigated the blue circle to the position where they would have deemed the target object to be second most likely to be (see Figure 2). Another difference was the selection of probe contexts: a total of 36 contexts were tested for recognition. Six of these were *repeated* contexts with one associated location, six were *repeated* contexts with two associated locations, six were *repeated* contexts associated with one hemifield, six were *novel* contexts shown once, and twelve were novel scenes that had not been shown before. The displays were again presented in randomized order. Participants were asked to indicate via mouse-click whether they had seen the scene before, not distinguishing between repeatedly shown scenes and scenes shown only once.

#### 3.1.4 Eye-movement analysis

The parameters for the eye-movement analysis in Experiment 2 were the same as in Experiment 1.

### 3.2 Results

#### 3.2.1 Repeated trials

The total proportion of trials that were repeated because of erroneous fixation on the initial fixation stimulus was 16.44% in the search task and 32.63% in the recognition test.

#### 3.2.2 Response accuracy

On average, participants failed to provide an answer before 20 s had passed in 5.44% of the trials (SD = 3.23%). Among the remaining trials, participants gave accurate answers (e.g., “T” when the target was a “T”) on average in 96.80% of the trials (SD = 2.78). The proportion of misses, but not accuracy, differed between conditions (for details, see Section 2.1.1 of the Supplementary material).

#### 3.2.3 Trial exclusion and missing data

Pooled over all participants, there were 11,520 trials in the search task. In 627 trials, no answer was given; these trials were excluded from analysis. An additional 352 trials were excluded because the participant gave an incorrect response. Based on the remaining trials in this speeded response task, each participant's mean manual response time and the corresponding standard deviation were calculated, and 331 trials in which the manual response time deviated from the mean by more than 3 standard deviations were excluded. Manual response time analysis was thus performed on 10,210 trials.

For the analysis of eye-movement data, among the 10,210 remaining trials, 128 trials needed to be excluded because no eye-tracking data were recorded. In another 320 cases, the number and duration of searching and responding fixations could not be determined because no fixation within the target interest area was registered. In a further 106 trials, the first fixation was also the first fixation of the last run on the target area of interest, so there were no searching fixation durations to be measured. Thus, analysis of the number of searching and responding fixations (including the cases in which there were zero searching fixations) was performed on 9,762 trials. Analysis of fixation duration was performed on 9,762 trials in the case of responding fixations and on 9,656 trials in the case of searching fixations.

#### 3.2.4 Manual response time

##### 3.2.4.1 Equality check in first block

In Experiment 2 as in Experiment 1, manual response times were not expected to differ between context conditions within the first block. After all, the manipulation only affected whether the context would be repeated in the upcoming blocks and whether it would always be repeated with the same, two different, or many different locations. In confirmation of this, context condition had no significant effect on manual response time within the first block, *F*_(3, 45)_ = 0.260, *p* = 0.854. A Bayesian ANOVA comparing the model including context condition as a predictor and participant as a random factor to the model only including participant as a predictor supported the absence of a context condition effect in the first block, BF_10_ = 0.115.

##### 3.2.4.2 Effect of context and epoch

**Figure 9:**
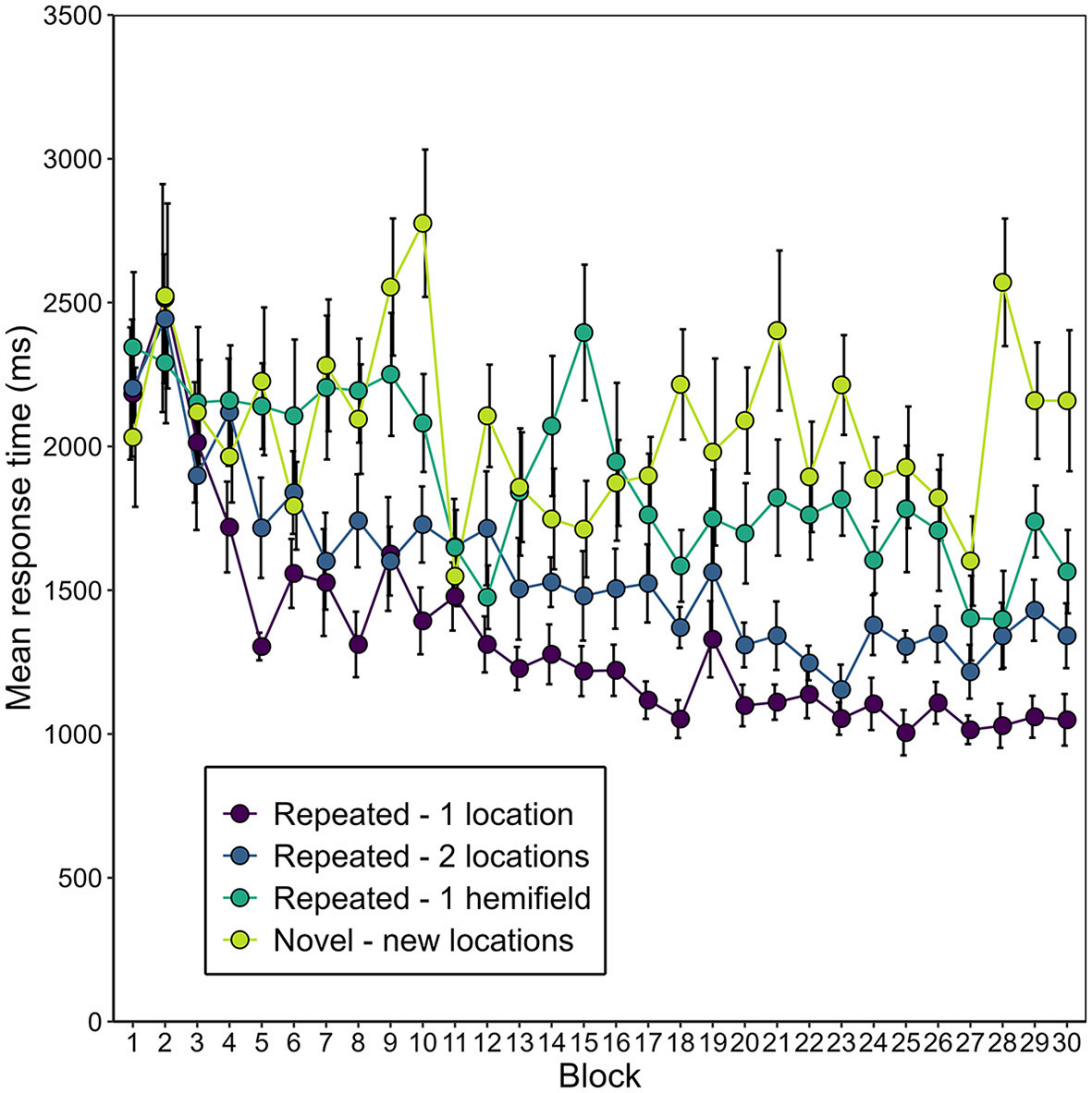
Mean manual response time (mean of the participants' average search time across scene identities) as a function of context (*repeated-1-location, repeate*d*-2-locations, repeated-1-hemifield*, or *novel*) and block in Experiment 2. Error bars represent standard errors for within-subjects designs (Morey, 2008).

Figure 9 depicts the development of manual response time over the blocks of the experiment, separately for the different conditions of repeated (1-location, 2-locations, 1-hemifield) and novel contexts. Again, to increase statistical power, manual response time was aggregated into epochs, this time six epochs, each containing five consecutive blocks. A 4 (context) × 6 (epoch) repeated-measures ANOVA revealed main effects of context, *F*_(3, 45)_ = 35.89, *p* < 0.001, $\eta_{\text{G}}^{2}$ = 0.248, and epoch, *F*_(1.56, 25.03)_ = 33.94, *p* < 0.001, ε_GG_ = 0.453, $\eta_{\text{G}}^{2}$ = 0.163. Notably, these factors interacted, *F*_(5.10, 76.47)_ = 2.95, *p* = 0.017, ε_GG_ = 0.340, $\eta_{\text{G}}^{2}$ = 0.053. Correcting for the multiple comparisons made on the manual response time data, we applied a Bonferroni-corrected alpha-level of α_corr_ = 0.00128. Context had a significant effect at every epoch except the first (see Supplementary Table 1). Conversely, epoch had a significant effect on manual response time in all contexts except the *novel* (*new locations*) contexts (see Supplementary Table 1).

**Figure 10:**
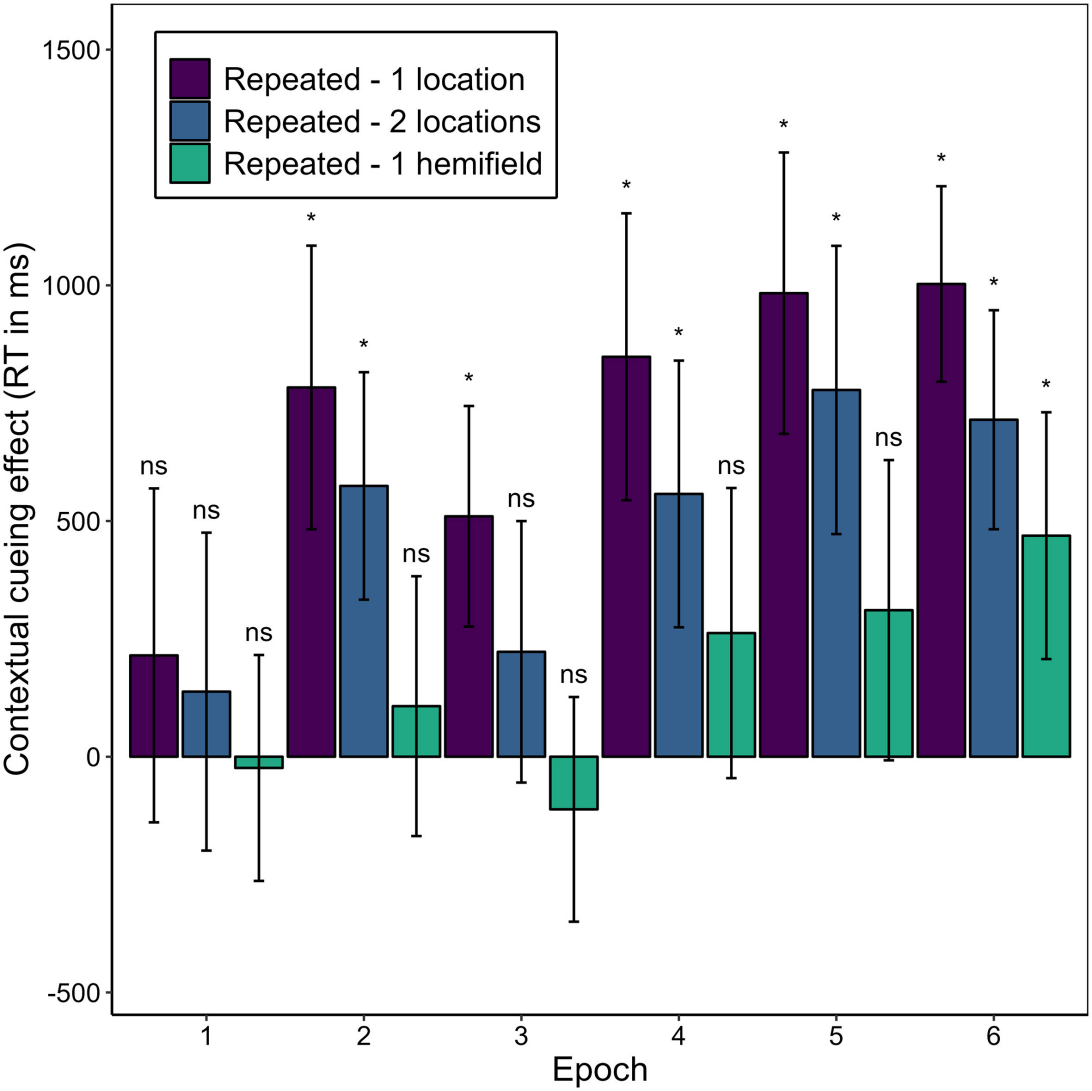
Contextual cueing effect as the mean difference in mean manual response times (RT) in *novel* contexts and *repeated* contexts with different numbers of associated target locations (*1-location, 2-locations, 1-hemifield*) for each epoch in Experiment 2. Error bars represent 95% confidence intervals for within-subjects designs (Morey, 2008). Asterisks (ns) denote cases in which mean manual response time did (not) significantly differ between the specified context condition and the *novel* context condition. For test statistics see Supplementary Table 2.

Of special interest was whether and, if so, when a contextual cueing effect arose for the different *repeated* contexts. Figure 10 illustrates the contextual cueing effect for each repeated context condition over the epochs. We expected that manual response time would decrease more heavily in *repeated-1-location* contexts compared to *novel* contexts early in the experiment, and that it would also decrease in *repeated*-2-location contexts, but later and to a less pronounced extent. We further expected that manual response time in *repeated-1-hemifield* contexts would decrease to the same extent as in *novel* contexts—yielding no difference between these contexts in any epoch. In *repeated-1-location* contexts, manual response time was significantly shorter than in *novel* contexts beginning with the second epoch. In *repeated-2-location*s contexts, manual response time was significantly shorter in the second epoch and then consistent from the fourth epoch (for *post-hoc t*-tests, see Supplementary Table 2). In *repeated-1-hemifield* contexts, manual response time differed significantly from response time in *novel* contexts in the sixth epoch.

These differences represent positive contextual cueing effects that developed over time through the repetition of contexts paired with one or two possible target locations, and that occurred somewhat later and in a weaker form in the case of two associated target locations. On average, manual response times in *repeated* contexts with one possible target location were 724 ms shorter (*SD* = 613 ms) than in *novel* contexts (*M* = 2,069 ms, *SD* = 554 ms). In *repeated-2-locations* contexts, they were on average 498 ms shorter (*SD* = 571 ms), and in *repeated-1-hemifield* contexts they were on average 169 ms shorter (*SD* = 568 ms).

##### 3.2.4.3 Manual response time by separate target locations

**Figure 11:**
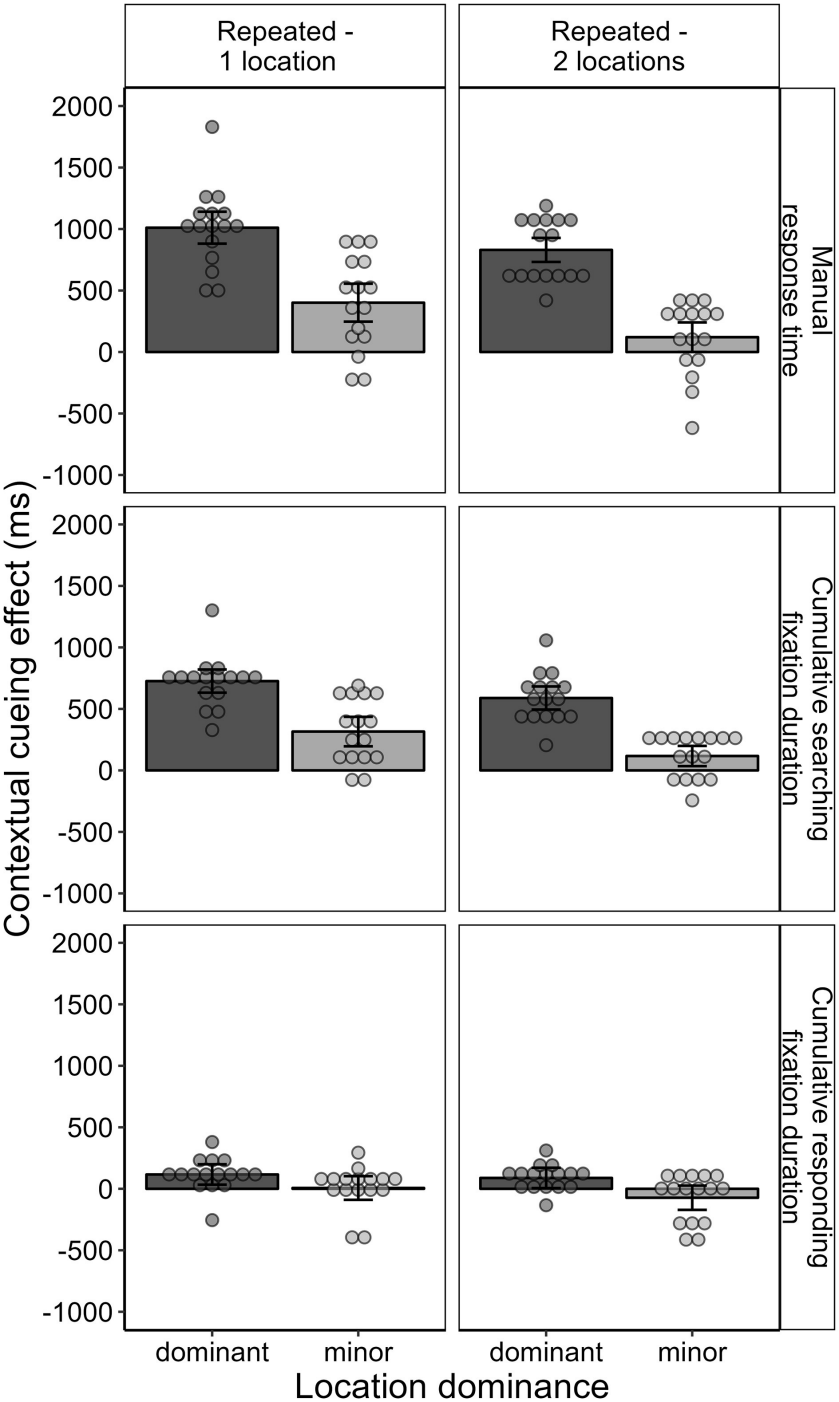
Contextual cueing effect across all blocks as the mean difference between *repeated* (*1-location* and *2-locations*) and *novel* contexts separately for dominant and minor target locations. The contextual cueing effect is depicted for manual response time, as well as for cumulative searching fixation duration and cumulative responding fixation duration.

The manual response time results for Experiment 2 so far revealed a contextual cueing effect for contexts associated with two target locations that was approximately 200 ms smaller than in contexts associated with only one target location, but still substantial, with the effect being in the range of half a second. In parallel with the analysis conducted in the study by Zellin et al. (2011), we examined whether the relatively small contextual cueing effect in contexts with two target locations was due to a missing or negative contextual cueing effect for the second location. In aid of this, we computed the mean contextual cueing effect separately for each context and target location. For each context, the target location with the larger contextual cueing effect was assigned to the “dominant” category of location dominance, and the target location with the smaller contextual cueing effect was assigned to the “minor” category. Across the whole experiment, as illustrated in Figure 11, the mean contextual cueing effect for both dominant and minor targets was positive. However, the contextual cueing effect was reliably different from zero only in the case of the dominant target, *t*_(15)_ = 13.176, *p* < 0.001, BF_10_ = 8,388,764, d_z_ = 0.928, and not for the minor target, *t*_(15)_ = 1.573, *p* = 0.137, BF_10_ = 0.709, d_z_ = 0.108. This suggests that only one of the target locations was cued by the scene context, while the other was not. However, costs for the second target locations were not observable either. A Bayesian *t*-test confirmed that there was stronger evidence for the contextual cueing effect being zero or positive than for a negative effect, BF_10_ = 11.457.

We applied a similar sorting procedure for the *repeated-1-location* contexts (cf. Zellin et al., 2011). For this condition, there were six contexts for each participant, each paired with only one target location. These contexts were randomly divided into three pairs. For every pair of contexts, the context with the larger contextual cueing effect was determined and assigned to the “dominant” category, while the other was assigned to the “minor” category. As expected, this procedure resulted in a larger positive average contextual cueing effect for the dominant scenes and a smaller positive average contextual cueing effect for the minor scenes. For statistical details and a comparison between the two major and the two minor contextual cueing between contexts with 1 and 2 target locations, please consult the Supplementary material.

**Figure 12:**
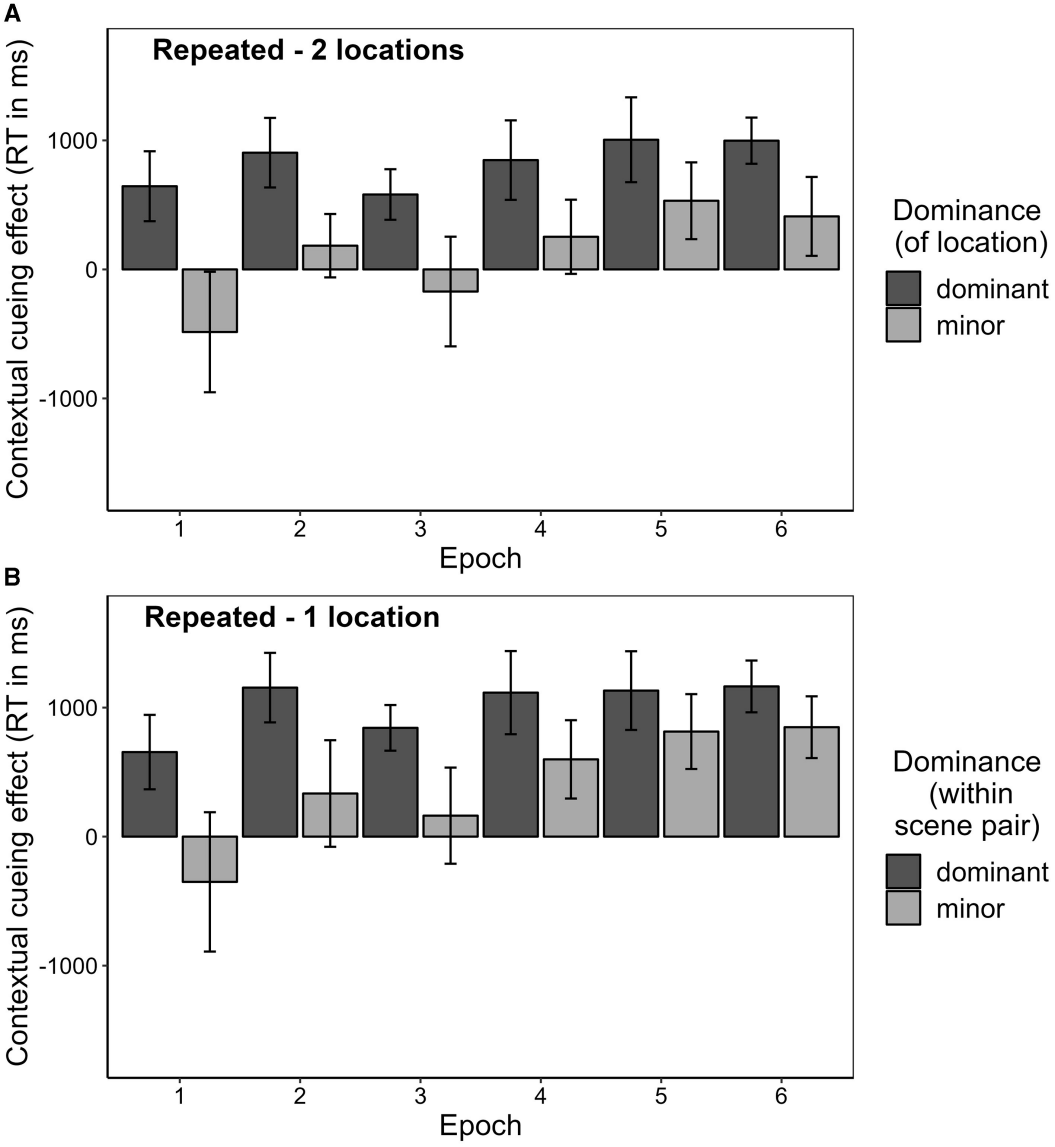
Contextual cueing effect on manual response time (in ms) over epochs seperately for dominant and minor targets. **(A)** *Repeated-2-locations*. Displays data from contexts associated with two locations, where for each context one location was assigned to the dominant category and the other to the minor category. **(B)** *Repeated-1-location*. Displays data from contexts associated with one location, where two scenes were always randomly paired and consequently, one was assigned to the dominant and the other to the minor category.

Since the contextual cueing effect is defined as an effect that develops over time (and cannot be present in block 1), we argue that it makes sense to examine not only the contextual cueing effects for the separate locations over the whole experiment, but also their development over time. As can be seen from Figure 12, in the first half of the experiment (epochs 1–3), the contextual cueing effect for the minor target was descriptively negative to small and positive, but did not differ significantly from zero in the case of either the *repeated-1-location* contexts, *t*_(15)_ = 0.283, *p* = 0.781, BF_10_ = 1.522, d_z_ = 0.276, or the *repeated-2-location*s contexts, *t*_(15)_ = −0.947, *p* = 0.358, d_z_ = −0.127. A Bayesian *t*-test provided some evidence that the contextual cueing effect for the minor target location might indeed be less likely to be positive or zero than negative (i.e., incurring contextual costs), BF_10_ = 0.240, as suggested by Zellin et al. (2011). However, for the second half of the experiment, the contextual cueing effect for the minor location was positive and significant for both *repeated-1-location* contexts, *t*_(15)_ = 5.846, *p* < 0.001, BF_10_ = 62,162.6, d_z_ = 1.173, and *repeated-2-locations* contexts, *t*_(15)_ = 5.082, *p* < 0.001, BF_10_ = 14,789.25, *d*_z_ = 0.410. As expected, for the dominant location the contextual cueing effect was significantly positive in both halves of the experiment in *repeated-1-location* contexts as well as *in repeated-2-locations* contexts. For the corresponding tests, please consult Supplementary Table 3.

###### 3.2.4.3.1 Exploratory description of dominant vs. minor locations

To provide a starting point for future research, descriptive statistics for features of the separate target locations are summarized in Table 2. Descriptively, the dominant target location was presented as the first target location (in the first block) equally as often as the minor target location. The dominant target location was not consistently the target more to the left. However, manual response times to the target when it was first found at the dominant location were significantly faster than manual response times when it was first found at the minor location. Additionally, on average, dominant target locations were significantly closer than minor target locations to the center.

**Table 2:** Exploratory measures describing dominant vs. minor target locations in the repeated-2-locations contexts in Experiment 2.

**Measure**		***M***	***SD***	***MDN***	***df***	***t***	***p***	***BF*_10_**	***d*_z_**
Proportion of scenes in which the dominant target was shown first		0.51	0.20	0.5					
Degrees visual angle by which the dominant target was to the left of the minor target		−−0.31	2.51	−−0.24					
Manual RT for first instance of finding the target (ms)	Dominant location	1,899	857	1,360	15	−5.05	< 0.001	208.57	−1.26
	Minor location	3,399	1,401	3,018					
Distance between target and screen center (°visual angle)	Dominant location	8.63	1.42	8.78	15	−2.77	0.014	4.07	−0.69
	Minor location	9.72	1.06	10.04					

#### 3.2.5 Searching and responding fixations

##### 3.2.5.1 Number of fixations

**Figure 13:**
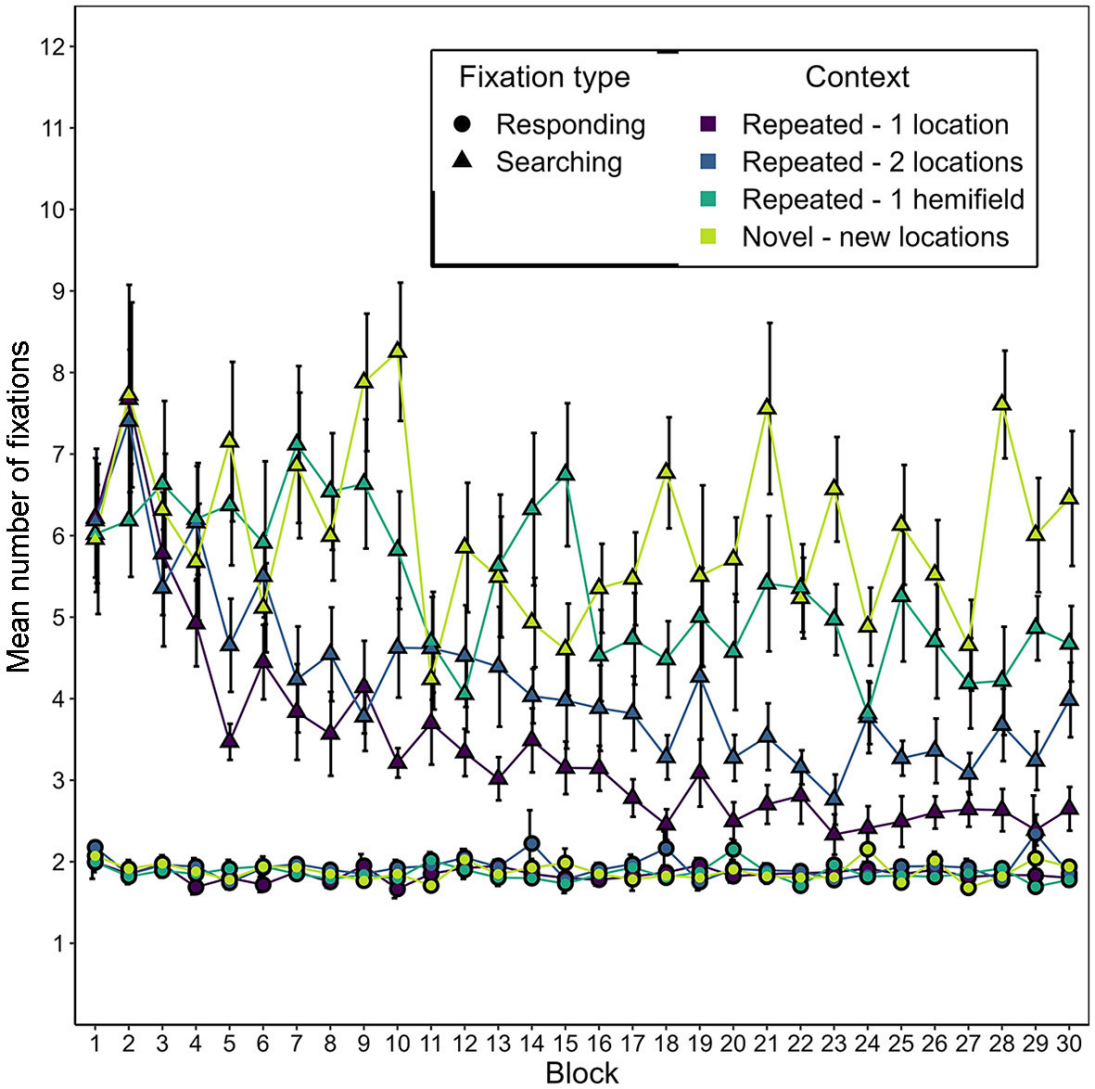
Mean number of responding and searching fixations as a function of block and context in Experiment 2. Error bars represent standard errors for within-subjects designs, calculated considering each fixation type seperately (Morey, 2008).

Figure 13 depicts the development of the mean number of responding and searching fixations over the course of the experiment, separately for the different conditions of *repeated* contexts and *novel* contexts. As in Experiment 1, the number of responding fixations was considerably lower in all contexts than the number of searching fixations. It was also striking again that the number of searching fixations, but not the number of responding fixations, decreased over time for *repeated* contexts. These impressions were confirmed by two 2-way repeated-measures ANOVAs with the factors of context and epoch for each fixation type. For the number of responding fixations, the effect of context did not survive Bonferroni correction for multiple testing, *F*_(3, 45)_ = 3.039, *p* = 0.039. Neither epoch, *F*_(2.9, 43.51)_ = 0.601, *p* = 0.613, nor the interaction of epoch and context, *F*_(4.9, 73.48)_ = 0.551, *p* = 0.734, had a significant effect on the number of responding fixations either. In contrast, context had a significant effect on the number of searching fixations, *F*_(3, 45)_ = 37.40, *p* < 0.001, $\eta_{\text{G}}^{2}$ = 0.289, as did epoch, *F*_(5, 75)_ = 28.77, *p* < 0.001, $\eta_{\text{G}}^{2}$ = 0.171. The interaction of context and epoch did not survive Bonferroni correction for multiple testing, *F*_(5.6, 83.97)_ = 2.51, *p* = 0.031, $\eta_{\text{G}}^{2}$ = 0.051. As in Experiment 1, this pattern of effects was grounded in a decrease in the number of searching fixations over the course of the experiment for the *repeated-1-location* contexts. Moreover, this decrease was also observed for *repeated-2-locations* contexts, but to a smaller degree (see Figure 13). This resulted in a significant contextual cueing effect on the number of searching fixations in *repeated* contexts associated with one or two target locations, starting from the second epoch (with a disruption in the third epoch for the *repeated-2-locations* contexts; see Supplementary Table 4). For the *repeated-1-hemifield* contexts, there was a significant effect of epoch, *F*_(5, 75)_ = 7.038, *p* < 0.001, $\eta_{\text{G}}^{2}$ = 0.195, but no contextual cueing effect on the number of searching fixations survived Bonferroni correction in any epoch (see Supplementary Table 4).

##### 3.2.5.2 Fixation durations

As in Experiment 1, searching fixation durations (*M* = 197 ms, *SD* = 37 ms) were significantly shorter than responding fixation durations (*M* = 287 ms, *SD* = 82 ms), as confirmed via a paired *t*-test, *t*_(15)_ = −10.97, *p* = < 0.001, *BF*10 = 835,231.5, *d*_z_ = −2.74. See Supplementary Figure 1 for the development of the mean median durations of responding and searching fixations over the course of Experiment 2.

As in Experiment 1, we expected responding fixation durations to decrease early in the experiment as a consequence of practice. Responding fixation durations differed significantly between epochs, *F*_(5, 75)_ = 5.00, *p* = 0.001, $\eta_{\text{G}}^{2}$ = 0.054. However, the effect of block on responding fixation durations during the first epoch was not significant, *F*_(1, 15)_ = 1.3, *p* = 0.271, $\eta_{\text{G}}^{2}$ = 0.042 (see also Supplementary Figure 1).

In contrast, context had no significant effect on responding fixation durations, *F*_(3, 43)_ = 0.28, *p* = 0.839. Responding fixation durations were comparably long in *repeated-1-location* contexts (*M* = 288 ms, *SD* = 53 ms) and in *novel* contexts (*M* = 289 ms, *SD* = 53 ms), *BF*_10_ = 0.12. The same was true for all other comparisons between contexts, *BF*_10_ = 0.12–0.17.

Context also had no significant effect on searching fixation durations, *F*_(1.87, 28.1)_ = 2.59, ε_GG_ = 0.624, *p* = 0.096, but epoch did have a significant effect, *F*_(3.2, 48.05)_ = 4.30, ε_GG_ = 0.641, *p* = 0.008, $\eta_{\text{G}}^{2}$ = 0.026. The interaction was not significant, *F*_(7.11, 106.68)_ = 0.494, ε_GG_ = 0.474, *p* = 0.492. Bayesian *t*-tests, however, indicated that fixation durations were longer in *novel* contexts (*M* = 201 ms, *SD* = 29 ms) than in *repeated-1-location* contexts (*M* = 192 ms, *SD* = 31 ms), *BF*_10_ = 178.60. In contrast, fixation durations in *repeated-2-locations* contexts (*M* = 198 ms, *SD* = 31 ms) seemed to be comparable to those occurring in *novel* contexts, *BF*_10_ = 0.641. For fixation durations in *repeated-1-hemifield* contexts (*M* = 197 ms, *SD* = 27 ms), the evidence was ambiguous as to whether these were shorter than fixation durations in *novel* contexts, *BF*_10_ = 2.47.

##### 3.2.5.3 Cumulative fixation durations in connection with manual response time

**Figure 14:**
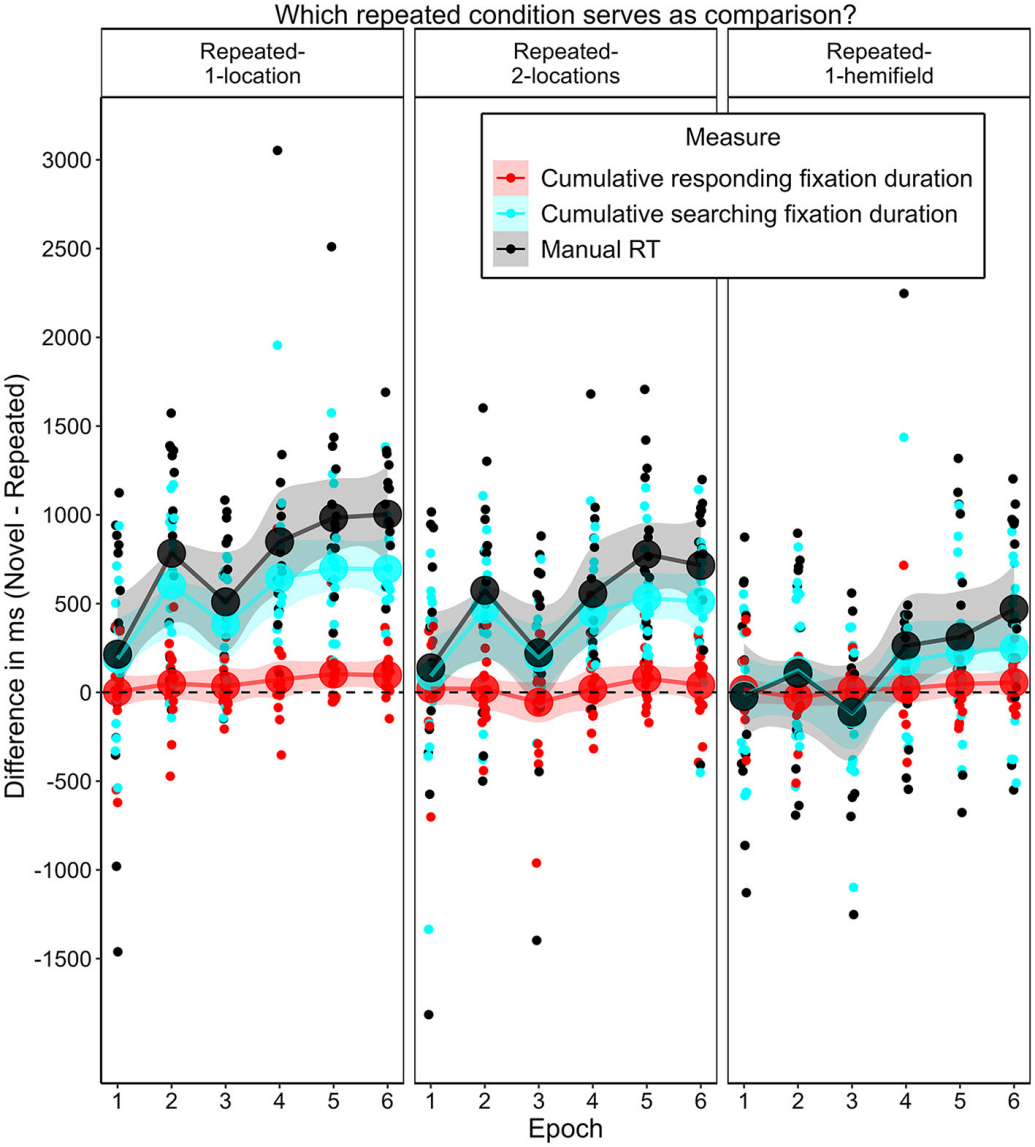
Development of the contextual cueing effect on manual response time and the two fixation types. Depicted is the mean difference in mean duration (manual response time, cumulative searching fixations, cumulative responding fixations) between *novel* and *repeated* contexts per epoch in Experiment 2, separately for every condition of *repeated* contexts serving as a comparison to performance in *novel* contexts.

As in Experiment 1, we also had reason to suspect in Experiment 2 that the observed contextual cueing effect was connected to searching fixations rather than to responding fixations. To determine whether there was support for the suspected connection between the contextual cueing effect and searching fixations, in parallel to Experiment 1, we set the manual response times in the context of the cumulative durations of the searching and responding fixations (see Figure 14). We could again confirm that (1) the summed cumulative fixation durations were always lower than or equal to the manual response time; and (2) the cumulative duration of searching fixations was significantly lower in *repeated* contexts than in the *novel* contexts, for *repeated* contexts with one possible target location, *t*_(15)_ = 12.53, *p* < 0.001, *BF*_10_ = 4,421,508, *d*_z_ = 3.133, and also for *repeated* contexts with two possible target locations, *t*_(15)_ =9.851, *p* < 0.001, *BF*_10_ = 225,871.2, *d*_z_ = 2.463. In *repeated-1-hemifield* contexts, participants did not spent significantly less time on searching fixations than in *novel* contexts, *t*_(15)_ = 1.756, *p* = 0.100, *BF*_10_ = 0.893, *d*_z_ = 0.439. Again, the cumulative duration of responding fixations did not differ between any of the *repeated* or *novel* contexts [*repeated-1-location*: *t*_(15)_ = 1.791, *p* = 0.093, *BF*_10_ = 0.935, *d*_z_ = 0.448; *repeated-2-locations*: *t*_(15)_ = 0.733, *p* = 0.475, *BF*_10_ = 0.323, *d*_z_ = 0.183; *repeated-1-hemifield*: *t*_(15)_ = 1.288, *p* = 0.217, *BF*_10_ = 0.516, *d*_z_ = 0.322]. Fulfilling criterion (3), and comparable to the contextual cueing effect on manual response times, the benefit in terms of the cumulative duration of searching fixations for *repeated* contexts significantly increased from epoch 1 to epoch 2. For contexts with one possible target location, the benefit increased on average by 415 ms (*SD* = 446 ms), *t*_(15)_ = 3.659, *p* = 0.002, *BF*_10_ = 18.561, *d*_z_ = 0.915, and for contexts with two possible target locations, the benefit increased on average by 377 ms (*SD* = 393 ms), *t*_(15)_ = 3.771, *p* = 0.002, *BF*_10_ = 22.561, *d*_z_ = 0.943. These increases in benefit were of comparable effect size to the augmentation of the contextual cueing effect on manual response time observed from epoch 1 to epoch 2 for *repeated* contexts associated with one location (*M* = 568 ms, *SD* = 541 ms), *t*_(15)_ = 4.128, *p* < 0.001, *BF*_10_ = 42.138, *d*_z_ = 1.032, or two locations (*M* = 436 ms, *SD* = 543), *t*_(15)_ = 3.160, *p* < 0.001, *BF*_10_ = 7.837, *d*_z_ = 0.790, which was the largest increment in contextual cueing. For *repeated-1-hemifield* contexts, there was no significant increase between epochs 1 and 2 in either the contextual cueing effect on manual response time, *t*_(15)_ = 1.101, *p* = 0.288, *BF*_10_ = 0.430 *d*_z_ = 0.275, or the benefit in terms of cumulative searching fixations, *t*_(15)_ = 1.365, *p* = 0.193, *BF*_10_ = 0.559, *d*_z_ = 0.341.

Again, for illustration, we calculated the correlation between participants' mean contextual cueing effect on manual response time per epoch and their mean contextual cueing effect on cumulative searching or responding fixation duration per epoch for each of the three *repeated* context conditions (see Table 3). In all context conditions, the manual contextual cueing effect correlated significantly more strongly with cumulative searching fixation durations than with cumulative responding fixation durations.

**Table 3:** Comparison of the mean of participants' correlations between the contextual cueing effect on manual RT and the effect on cumulative duration of responding vs. searching fixations (epoch averages) by condition.

**Condition serving as comparison for contextual cueing effect**	**Correlation of manual RT with**	***n***	***M (of r)***	***SE***	**95% CI**	***df***	***t***	***p***	***BF*_10_**	***d*_z_**
**Experiment 1**										
Repeated – 1 location	Cumulative searching fixation duration	17	0.83	0.07	[0.67, 0.98]	16	1.83	0.087	0.969	0.44
	Cumulative responding fixation duration	17	0.5	0.14	[0.20, 0.81]					
**Experiment 2**										
Repeated – 1 location	Cumulative searching fixation duration	16	0.89	0.03	[0.83, 0.95]	15	4.27	< 0.001	53.96	1.07
	Cumulative responding fixation duration	16	0.40	0.12	[0.15, 0.66]					
Repeated – 2 locations	Cumulative searching fixation duration	16	0.84	0.04	[0.75, 0.93]	15	4.16	< 0.001	44.92	1.04
	Cumulative responding fixation duration	16	0.52	0.07	[0.38, 0.66]					
Repeated – 1 hemifield	Cumulative searching fixation duration	16	0.90	0.03	[0.84, 0.95]	15	6.39	< 0.001	1, 854.18	1.59
	Cumulative responding fixation duration	16	0.51	0.06	[0.39, 0.63]					

##### 3.2.5.4 Cumulative fixation durations by separate target locations

In a previous section, we aimed to determine whether pairing contexts with two target locations instead of one indeed results in manual response time costs in the process of finding the less favored location compared to baseline. Although the possibility could not be excluded that, in the first half of the experiment, search was slower for minor targets in *repeated* contexts than for targets in *novel* contexts, a stable benefit in manual response time developed even for the minor of two targets during the course of the second half of the experiment. Against the background in which, as established in the preceding paragraph, the contextual cueing effect observed in manual response time corresponds to the cumulative duration of searching fixations but not responding fixations, we aim now to differentiate the respective contributions of searching and responding fixations to the contextual cueing effects for dominant and minor locations. Analogous to the contextual cueing effect averaged over dominant and minor locations, any positive contextual cueing effect for the dominant or minor locations should correspond to a positive contextual cueing effect on cumulative searching fixation durations. Regarding the question of what drives the difference in the size of the contextual cueing effect between dominant and minor locations, at least two scenarios are imaginable. One possibility is that the reduced degree of contextual cueing for the minor location is because the apparent improvement in guiding of selective attention through contextual cueing does not occur to the same extent for the minor location, be this because of less effective learning or because every time the context appears attention is first guided to the dominant location. In this case, the contextual cueing effect on cumulative searching fixation durations for the dominant and minor location should follow the same pattern as the effect on manual response time, while no effect of context on cumulative responding fixation durations should be visible. Alternatively, attentional guidance to the minor location might improve as greatly as it does for the dominant location, but with identification of and initiation of the response for the less favored target location taking longer. In this case, cumulative searching fixation durations for the dominant and the minor location should show a comparable contextual cueing effect, but cumulative responding fixations should show a negative contextual cueing effect for the minor location.

As we have established in a previous section that it is crucial to consider the contextual cueing effect for major and minor locations over time, we here report the results separately for each half of the experiment. The separate contextual cueing effects on cumulative searching and responding fixation durations for dominant and minor target locations are summarized in Figure 11. Table 4 shows the corresponding statistics in the form of paired *t*-tests for those effects. These tests revealed that only cumulative searching fixation durations, and not cumulative responding fixation durations, differed between *repeated-2-locations* contexts and *novel* contexts. In the case of *repeated-1-location* contexts, the contextual cueing effect for the minor target was only significant in the second half of the experiment. While cumulative responding fixation durations never differed significantly between *repeated* and *novel* contexts, calculation of a Bayes factor indicated that there was some evidence for a negative contextual cueing effect on cumulative responding fixation durations in the second half of the experiment, BF_10_ = 0.23. However, even in this case the effect would be very small, *d*_*z*_ = –0.117.

**Table 4:** *T*-tests of the contextual cueing effect on cumulative duration of searching and responding fixations by experiment half and dominance of target location in Experiment 2.

**Measure**	**Experiment half**	**Context condition**	**Dominance**	**n_1_**	**n_2_**	**t**	**df**	***p***	***d_*z*_***	***BF_10_***
Cumulative searching fixation duration	1	Repeated-1-location	Dominant	16	16	12.276	15	< 0.001	0.837	6.32 × 10^8^
			Minor	16	16	0.680	15	0.507	0.366	2.76 × 10^0^
		Repeated-2-locations	Dominant	16	16	6.340	15	< 0.001	0.789	1.51 × 10^5^
			Minor	16	16	−0.774	15	0.451	−0.087	3.10 × 10^−1^
	2	Repeated-1-location	Dominant	16	16	8.733	15	< 0.001	1.201	6.95 × 10^6^
			Minor	16	16	6.455	15	< 0.001	1.205	1.84 × 10^5^
		Repeated-2-locations	Dominant	16	16	11.255	15	< 0.001	1.096	1.94 × 10^8^
			Minor	16	16	4.068	15	0.001	−0.087	3.79 × 10^1^
Cumulative responding fixation duration	1	Repeated-1-location	Dominant	16	16	1.965	15	0.068	0.237	2.24 × 10^1^
			Minor	16	16	−0.584	15	0.568	−0.029	4.20 × 10^−1^
		Repeated-2-locations	Dominant	16	16	1.509	15	0.152	0.199	1.03 × 10^1^
			Minor	16	16	−1.213	15	0.244	−0.198	1.60 × 10^−1^
	2	Repeated-1-location	Dominant	16	16	3.932	15	0.001	0.403	8.24 × 10^2^
			Minor	16	16	1.028	15	0.320	0.255	4.74 × 10^0^
		Repeated-2-locations	Dominant	16	16	3.502	15	0.003	0.369	3.71 × 10^2^
			Minor	16	16	−0.968	15	0.348	−0.117	2.30 × 10^−1^

The *t* statistic represents paired *t*-tests between the specified context condition and the *novel* context condition. For the *novel* condition contexts, no distinction between dominant and minor locations was made, so all data points from the first or second half of the experiment were used irrespective of whether the comparison was to dominant or minor locations. The reported Bayes factor BF_10_ tests the hypothesis that δ ≥ 0 against the hypothesis that δ < 0.

#### 3.2.6 Recognition performance

##### 3.2.6.1 Scene recognition

**Figure 15:**
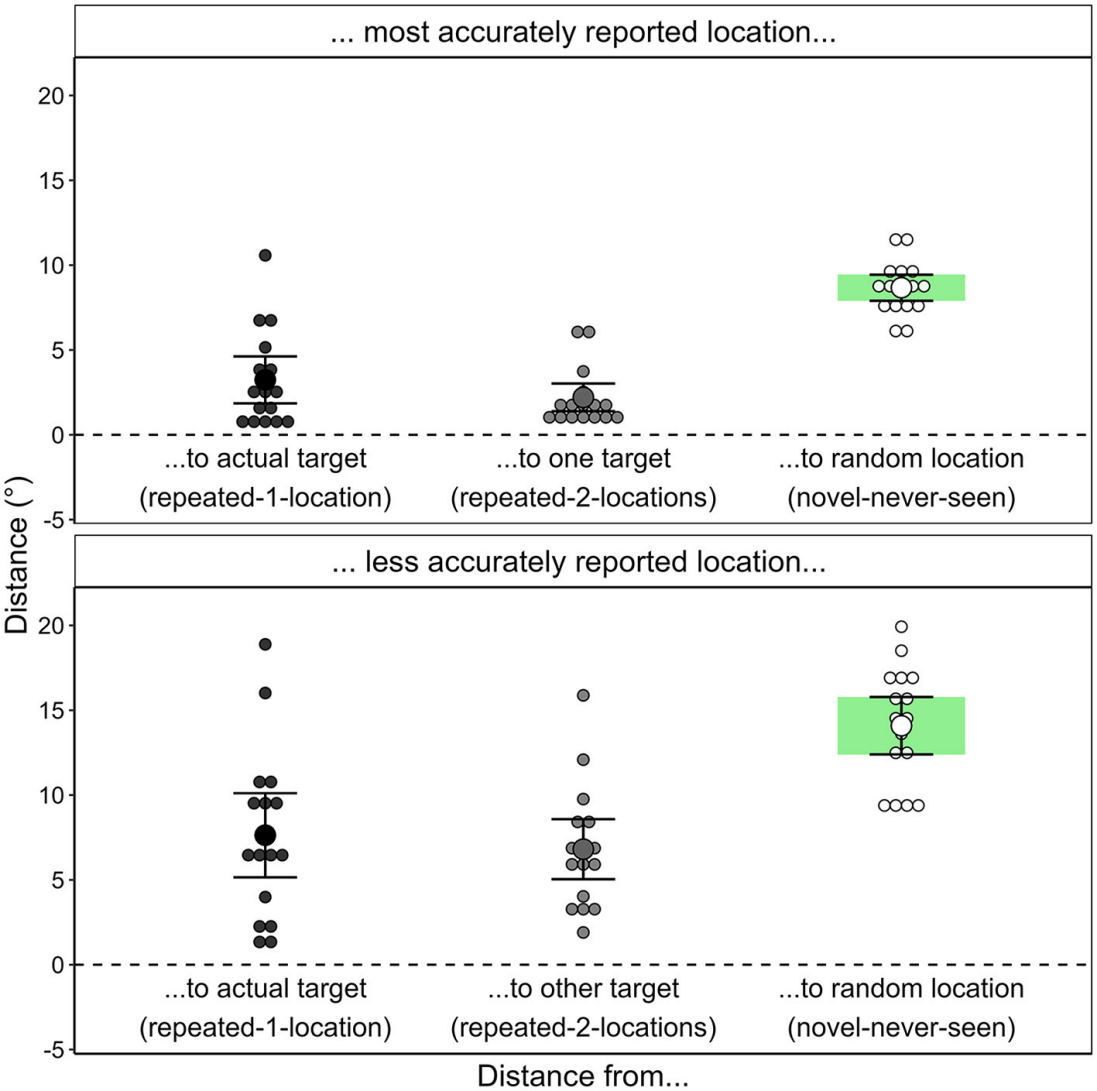
Accuracy of participants' target location generation in the *repeated-1-location, repeated-2-locations*, and *novel* conditions in Experiment 2. This graph depicts the mean placement error by context condition for each participant, calculated as the distance in degrees of the visual angle between the generated locations and the actual or a random dummy target location.

Participants' scene recognition performance in Experiment 2 is illustrated in Figure 15. On average, participants reported remembering 95.49% of the *repeated* scenes (pooled over *1-location, 2-locations, 1-hemifield*), 53.13% of the scenes shown once in the search task, and 7.81% of the novel scenes (i.e., false alarms). A Wilcoxon signed-rank test showed that the percentage of repeated contexts correctly recognized as old (*Mdn* = 100%) was significantly higher than the false alarm rate (*Mdn* = 0%; *z* = −3.5239, *p* < 0.001, *r* = −0.626), as was the percentage of contexts shown once that were recognized as old (*Mdn* = 50%, z = −3.522, *p* < 0.001, *r* = −0.623).

##### 3.2.6.2 Accuracy of location generation

As in Experiment 1, the accuracy of the participants' memory for the target position was operationalized as the distance between the location generated in the recognition test and the actual location or a dummy location (see Figure 15). Since participants were asked to generate two locations for every scene, for analysis we used the generated location that was closest to the actual/dummy target. However, for the *repeated* contexts that were associated with two locations, we also considered the second, less accurately generated target location, because we suspected it to represent the participant's memory for the location of the other target associated with the context. Participants' mean accuracy in generating the target location for *repeated-1-location* contexts (*M* = 3.24°, *SD* = 2.82°), measured as the distance from the nearest generated location to the actual target position for the corresponding scene, was significantly better than their accuracy for the *novel* (never-seen) contexts (baseline condition, *M* = 8.67°, *SD* = 1.57°), where accuracy was measured as the distance between whichever of the two generated locations was closer to the dummy location (a randomly generated location for each trial in the area of possible target locations), *t*_(16)_ = −6.92, *p* = < 0.001, *BF*_10_ = 4,212.65, *d*_z_ = −1.73. For *repeated-2-locations* contexts, not only was participants' accuracy for the most accurately generated location (*M* = 2.20°, *SD* = 1.66°) better than their accuracy in the baseline condition, *t*_(16)_ = −10.85, *p* = < 0.001, *BF*_10_ = 726,805.20, *d*_z_ = −2.71, but their accuracy for the other location was also better than their accuracy in the baseline condition, *t*_(16)_ = −6.68, *p* = < 0.001, *BF*_10_ = 2,900.06, *d*_z_ = −1.67. To take into account the selection effect of selecting the “worse” generated location, we also compared this to the less accurately generated location in the *novel* (never seen) contexts.

## 4 General discussion

This study examined whether a contextual cueing effect would arise for two targets within a natural scene. Additionally, we investigated whether differences in the contextual cueing effect between contexts associated with one and two target locations, as well as differences between the more and the less favored of two cued target locations, can be assigned to (which of) either of two consecutive phases of the visual search process, namely, to searching and/or responding fixations.

Experiment 1 replicated a profound contextual cueing effect in natural scene contexts. Additionally, eye-tracking data delivered evidence that this effect was grounded in more efficient attentional guidance in the *repeated* vs. *novel* contexts. As expected, Experiment 2 provided further evidence of the same profound contextual cueing effect, and it did so also for natural scene contexts that were associated with two possible target locations. Importantly, while two-target-location cueing resulted in one major and one minor target location, by the second half of the experiment the contextual cueing effect was positive and substantial in size for both of them. As in Experiment 1, the contextual cueing effect seemed to be grounded in a more efficient attentional guidance phase, as indicated by a reduction in the number and cumulative duration of searching fixations. Notably, this also applied to two-target-location contexts. Likewise, the differing sizes of the contextual cueing effect for minor and major target locations also appeared to be due to more efficient guiding toward the major target locations than to response-related processes of visual search.

### 4.1 Contextual cueing for multiple locations in natural scenes vs. arrays

Can natural scenes cue multiple locations? Yes. A contextual cueing effect for two-target-location contexts was clearly visible in Experiment 2. Comparable to previous studies (e.g., Kunar et al., 2005; Zellin et al., 2011; Vadillo et al., 2021), the observed contextual cueing effect for two-target-location contexts was smaller in magnitude compared to one-target-location contexts. However, with an effect size of 498 ms (vs. 724 ms or 737 ms), this was still a substantial effect, presumably owing to the natural type of context presented.

Zellin et al. (2011) suggested that a contextual cueing effect for two-target contexts only represents a contextual cueing effect for one well-learned location averaged with a negative contextual cueing effect for the less favored location. This would be compatible with the finding by Yang et al. (2021) that when a contextual cueing effect has already developed for one location, participants do not develop a contextual cueing effect for a new consistent target location introduced in the same context. In contrast, however, we were able to show that by the second half of the experiment the contextual cueing effect for both possible target locations was clearly positive. This shows that it is indeed possible to prioritize two locations simultaneously contingent on one scene context.

Why might our results differ from previous findings? First, interleaved learning of both target locations as opposed to consecutive massed learning (cf. Yang et al., 2021) might be a prerequisite for this flexible learning outcome. However, in this regard we used the same method as Zellin et al. (2011). Therefore, one suggestion for an explanation for the differences could be that there simply were not enough instances (i.e., blocks of trials; Kunar et al., 2005) for possible learning of an additional second target location to compensate for the costs otherwise incurred as a result of the other location already having been learnt. Even focusing on the learning aspect only, it seems plausible that alternating learning of two associations will take longer than consistent learning of one (Schneider and Shiffrin, 1977). In our study, 16 opportunities (32 blocks of trials) for each context–target location association were sufficient to reject the possibility of a negative contextual cueing effect for the minor location; however, 20 opportunities in the study by Zellin et al. (2011) did not allow this conclusion. Possibly, since contextual cueing seems to arise more quickly and with substantially greater magnitude for natural scenes (Brockmole and Henderson, 2006b), even more blocks could have been needed in the configurational contextual cueing paradigm. Evidently, Zellin et al. (2011) did not investigate the temporal development of the contextual cueing effect for the two locations separately, which was what allowed us to see a clear positive contextual cueing effect for the minor target location in the second half of our experiment. Potentially, their data would have shown a similar pattern.

Still, it could also be the case that natural scene stimuli activate different search mechanisms that allow cueing of two locations in parallel, which does not happen for configurational stimuli. Configurational contextual cueing probably has a strong local constraint (Brady and Chun, 2007), although it can also be observed for the global configuration (e.g., Zheng and Pollmann, 2019). In comparison, scene-based contextual cueing is considered to depend mainly on the global context (e.g., Brockmole et al., 2006; Brooks et al., 2010; Castelhano et al., 2019). It seems plausible that natural scene stimuli might not only encourage global vs. local contexts to guide search, but also encourage more distributed attention. Distributed attention, as opposed to focused attention, has been shown to be more flexible in, e.g., incorporating changes in the target location (Zinchenko et al., 2020), presumably because attending to the global distractor configuration means attending to something constant that canact as a reliable contextual cue even for a new target location. To learn that one context is associated withtwo possible target locations, it might have been similarly helpful that natural scenes or their gists were usedas global cues, rather than associating two different local contexts with each target location.

Thus, the present results confirm previous findings of positive contextual cueing effects for multiple target locations and suggest that the idea of lasting contextual costs for the second target location should be rejected. This might be either specific to natural scene contexts or generally applicable provided that the environment offers enough opportunities to build all associations. At the same time, the smaller contextual cueing effect for two-target contexts is consistent with an attentional guidance account (Brady and Chun, 2007).

### 4.2 Processing locus of contextual cueing for one- and two-target-location contexts in natural scenes vs. arrays

Compatible with our results, Wang et al. (2020) argued that the contextual cueing effect that they observed for contexts associated with two (or four) target locations was present for both locations. Still, their interpretation of these results is not transferable to our data. As mentioned in the introduction, Wang et al. (2020) presumed that in cases of more than one associated target location, it is no longer the attentional guidance phase that is affected by contextual cueing. Having observed a lack of much difference in magnitude between contextual cueing effects for two and four possible target locations, the authors concluded that facilitation of decision or response selection processes might be responsible for the benefits. We explicitly investigated this question of the processing locus of the contextual cueing effect for multiple target locations. We analyzed the number and duration of searching vs. responding fixations as representatives of an early attentional guidance phase vs. a later phase of response selection processing. As a first step, we established that these classes of fixations are qualitatively different. Evidently, searching fixations were greater in number than responding fixations, but the durations of the latter were approximately 100 ms longer than those of the former. In both experiments, the number of searching fixations, but not the number of responding fixations, exhibited a contextual cueing effect. In a three-step argument, we demonstrated how the contextual cueing effect on cumulative searching fixations parallels the contextual cueing effect on manual response time. This led us to the conclusion that the response time benefit in repeated contexts emerges in the searching phase, making the case for attentional guidance vs. response facilitation. Importantly, this was valid for the contexts associated with one target location as well as those associated with two.

Perhaps, again, our results might be specific to natural scene contexts, and the mechanisms may differ in studies such as the one by Wang et al. (2020). Decision and response processes that might be facilitated through contextual cueing might only be crucial in the case of configurational contexts (arrays). One indication is that in configurational contexts, response times usually decrease over blocks, not only for the repeated configurations but also for novel ones. This is commonly interpreted as a procedural learning effect of practice (cf., Jiang and Sisk, 2019; Yang et al., 2021). However, in scene-based contextual cueing, the effect of epoch is often only visible for repeated contexts (e.g., Experiment 1 of the present study, Brockmole and Henderson, 2006b). Therefore, repeating targets in a specific location within a configuration might induce improvements in response times due to perceptual learning, which might be irrelevant in natural scene contexts for which humans have high existing expertise.

Alternatively, the findings by Wang et al. (2020) might also be explained by other mechanisms. Wang et al. (2020) pointed out that the type of contextual cueing in their study was somewhat different from the usual type. What was particular about it was the fact that a repeated context that had four locations associated with it shared these associated locations with four other repeated contexts. If participants had somehow extracted this regularity, they might have made use of trials of other contexts from the same context quartet to strengthen the learning of the four associated locations. This could potentially explain why they even observed that the search benefit in the contexts paired with four locations was greater after four opportunities for each relative context–target location association (blocks 5–16) than in the contexts paired with only one location (blocks 2–4). In this way, the results could again be explained by attentional guidance, but on the basis of more complex underlying associations.

Another mechanism that would also not necessarily assume differences between the number of target locations would be distractor suppression. Repeated presentation of search contexts might convince participants that distractor locations are unlikely to contain the target and can therefore be ignored (cf., Brady and Chun, 2007; Failing et al., 2019; Vadillo et al., 2021). This would also lead to a shorter early searching phase, just as we observed. However, distractor suppression is less likely to be responsible for the present results. Targets were superimposed on photographs and could appear anywhere, independent of the locations of natural objects in the scene, so ignoring distractor locations would not have been very productive in helping targets to stand out. Furthermore, distractors are abundant in natural scenes and ignoring them all does not appear to be an efficient processing strategy for the human brain to employ, even though distractor suppression might be useful in situations with few and predictable distractor locations. Moreover, should distractor suppression have produced the present contextual cueing effects, this should have produced a contextual cueing effect for those repeated contexts that were less predictive of the target locations as well, being restricted only with respect to the hemifield. Indeed, in the last epoch, a contextual cueing effect also arose for this condition. As with the other conditions, this effect seemed to be located in the generation of fewer searching fixations, but the evidence was not clear enough to draw a firm conclusion. As mentioned, the contextual cueing effect for repeated-1-hemifield contexts emerged later and with smaller magnitude than the effect observed in the one- and two-target-location conditions. We do not know whether the hemifield regularity or the repetition alone fueled the development of this effect, and it is an open question for further research whether this hemifield effect is attributable to the same mechanism(s) that generate contextual cueing effects in the one- and two-target-location conditions. A weaker contextual cueing effect if only parts of the mechanism are at work would be compatible with the conclusion drawn by Beesley et al. (2015). Although they showed that a model that learns distractor–distractor associations additionally to distractor–target associations is a better fit to the empirical data, they concluded that distractor–target associations remain crucial for the development of a (large) contextual cueing effect. When sticking with an attentional guidance account of contextual cueing, the observation of a contextual cueing effect in the repeated-1-hemifield condition represents an initial indication that a model in which only specific target locations are cued by a context (cf. Brady and Chun, 2007) would have to be adjusted so that cueing of regions could also be expected.

To sum up, the present results strongly suggest an important role of guidance by context–target–location associations in the contextual cueing effect. This aspect of the effect seems to play an especially large role in natural scene contexts, but might also explain findings in configurational contextual cueing.

### 4.3 Potential limitations

In contextual cueing paradigms, a fixed set of target locations is usually used for repeated and novel contexts, such that each target location occurs equally frequently in order to prevent location-probability effects (Miller, 1988; Geng and Behrmann, 2002; Jiang et al., 2013). In adapting the paradigm of Brockmole and Henderson (2006b), we deviated from this and drew target locations randomly while making sure that they occurred equally often within each hemifield. This meant that we could not control location probability as thoroughly as the fixed location sets, and by accident may have allowed location-probability effects to occur. However, across all trials, targets' locations were likely to be scattered uniformly across the display. Additionally, participants' ability to generate the target location for repeated contexts could be taken as an indicator against the possibility that observed contextual cueing effects could be alternatively explained by location-probability cueing. After all, the test subjects were able to generate a fairly exact location in accordance with the context shown during the recognition test. If the response time benefit were solely due to location probability, then this would only result in such accurate location generation if the locations were similar for all contexts shown to a given participant, which was not the case. As mentioned in the stimulus section, the median distance from the target locations (11.96° visual angle in Experiment 1) within one participant's trials was much greater than the accuracy with which they generated the context-contingent target locations.

It has been pointed out that the amount of regularity in an environment seems to critically influence which information the visual system will detect and potentially learn (Zang et al., 2018; Zinchenko et al., 2018). In our Experiment 2, in a within-subjects design, we examined four conditions in parallel; only one of these four conditions did not include a regularity of association between context and target location. Experiencing the strict contingency of the context and the associated target location in the *repeated-1-location* condition might have prepared the visual system to detect similar regularities across all conditions, boosting the effect on attentional guidance for the r*epeated-2-locations* and perhaps also for the *repeated-1-hemifield* condition. In future research, it might be interesting to confirm whether the contextual cueing effect for multiple-target-location contexts does also lie in attentional guidance in the absence of any other contexts bearing associative regularities.

Although the contextual cueing effect observed in our experiments resembles those observed by, e.g., Brockmole and Henderson (2006a,b) in terms of its size, the results of this experiment might not be generalizable to all naturalistic scene stimuli. For example, the large variance in manual response times, especially for novel contexts, illustrates the fact that search time itself is highly dependent on the specific scene stimulus, and therefore search benefits obtained through learning are also constrained in their size by the nature of the specific stimulus. On the one hand, more homogenous scene stimuli might be attractive for replication and generalization of these results. On the other hand, even less homogenous natural scene stimuli and more naturalistic target stimuli are needed to confirm the relevance of the findings for visual search under real-life conditions.

### 4.4 Outlook

This study was able to show that when a natural scene context is predictive of not only one but two likely target locations, learning can occur to guide attention toward both target locations. However, how exactly prioritization of one or the other possible target location unfolds over time is unclear. We speculate that the difference between major and minor target locations in terms of the magnitude of the response time benefit might have a real correspondence in terms of how the target locations are represented in memory (Pollmann and Schneider, 2022). What determines a possible hierarchy of target locations for a specific context and the way they are commonly or sequentially utilized in search might be an important topic for future studies. As a starting point, our explorative analysis of the dominant and minor target locations revealed that almost as many second-presented as first-presented target locations became the dominant target location. Pollmann and Zheng (2023) showed that a larger contextual cueing effect develops for targets on the right vs. the left half of the display. They argue that, due to an initial leftward bias, participants already respond quickly to targets in the left half of the display, so that there is not as much opportunity to improve as with targets in the right half of the display. Since the two possible target locations for each scene in our study were on the same side of the screen, an effect of screen side could not be tested for directly. However, there was little evidence that the dominant target was generally more to the left or more to the right. One initial piece of information could be the fact that, for dominant target locations, the average response time for the first instance of finding the target was lower than in the case of minor target locations. On the one hand, this could simply mean that the dominance of a target location might just be an artifact of the assignment process, with easy search difficulty being confused with dominance/strong learning. On the other hand, it could indicate that (incidental) fast search promotes stronger learning. This should be experimentally tested. Another finding that should be experimentally tested is the tendency for the dominant target to be more central than the minor target. When humans view stimuli on a computer screen, they typically show a bias toward fixating on the center (Tatler, 2007). Because of this center bias, it might have been easier for participants to detect more central targets. A systematic effect of target eccentricity on dominance among the contextually cued locations and its connection to the aforementioned link between fast search and learning should be examined directly in future studies.

Another open question is to what extent visual search benefits from associating more than two targets with a particular natural scene context. The present study is in line with the expectation that contextual cueing effects for three, four, or more locations might be possible. However, the findings also support the expectation that contextual cueing effects are not equally sized for all associated target locations. As speculated upon above, the observed guidance benefits could be understood as originating in one-to-one associations between scene identity and a specific location within that scene. Guidance according to learnt relevant locations might, with high likelihood, unfold in a sequential manner, allowing only smaller benefits for target locations lower in the hierarchy. Studies on visual search in the case of foraging could be informative in this respect.

### 4.5 Conclusion

In conclusion, the present study showed that natural scenes can indeed cue attention in cases of multiple target locations. Although a benefit was only observed for one target location during the first half of Experiment 2, during the second half, contextual cueing effects were clearly positive for both possible target locations. Costs for trials in which the target is shown at the less favored location do not have to be interpreted as costs specific to this location. Rather, the build-up of the associations probably does not happen simultaneously, resulting in some misguidance to the already-learnt location when the second target location has not yet been learnt. Detailed eye-movement analysis confirmed previous findings that contextual cueing affects searching fixations before the target is found, rather than responding fixations made during preparation of a response. Therefore, it is likely that contextual cueing in natural scenes is driven by attentional guidance based on associations between global scene representations and (a) specific target location(s). Notably, the same process seems to have benefitted from two-target location cueing and also to be responsible for the difference between major and minor locations. The specificity of these effects to natural scene contexts has been discussed and further research issues presented.

## Data availability statement

The preprocessed data, analysis code, and experiment code (https://osf.io/pmeaf/) are available on the Open Science Framework. The raw data supporting the conclusions of this article will be made available by the authors, without undue reservation.

## Ethics statement

The studies involving humans were approved by Bielefeld University's Ethics Committee (EUB). The studies were conducted in accordance with the local legislation and institutional requirements. The participants provided their written informed consent to participate in this study.

## Author contributions

JA: Conceptualization, Formal analysis, Investigation, Methodology, Software, Visualization, Writing – original draft, Writing – review & editing. WS: Conceptualization, Methodology, Resources, Supervision, Writing – review & editing. CP: Methodology, Software, Supervision, Validation, Writing – review & editing.
