# Supplementary material for: Can natural scenes cue attention to multiple locations? Evidence from eye-movements in contextual cueing
Source: Front Cognit. 2024 Mar 13;3:1352656. doi: 10.3389/fcogn.2024.1352656 (PMC13281219; doi:10.3389/fcogn.2024.1352656)
Supplement: Supplementary file 1 [file Data_Sheet_1.docx]

Supplementary Material

# Experiment 1

## Supplementary results

### Response accuracy.

The proportion of trials in which no answer was made before 20 s had passed ranged among participants from 0.78% to 20.31% (*M*= 6.76%*,* *SD* = 4.79%). Neither the proportion of misses nor accuracy differed between conditions. Detailed information can be found in the Supplement. The proportion of misses in the *Novel – new locations* condition (*M*= 8.00%*,* *SD*= 4.14%) was not significantly different to the proportion in the *Repeated – 1 location* condition (*M* = 5.51%*,* *SD* = 7.24%), *t*(16) = ‑1.486, *p*= 0.157, *d*_z_ = 0.360, *BF*_10_ = 0.629. Neither did the proportion of response errors (e.g., “T” when the target was an “L”), differ between *novel* (*M*= 3.03%*,* *SD*= 1.63%) and *repeated* contexts (*M*= 3.26%*,* *SD*= 2.33%), *t*(16) = 0.397, *p*= 0.697, *d*_z_ = 0.096, *BF*_10_= 0. 267. Referring to all trials (no exclusions made, i.e. including trials with incorrect discrimination and misses), accuracy ranged between 73.44 % and 96.09% (*M* = 90.10%, *SD*= 5.73%). When referring only to trials with timely responses, accuracy ranged between 92.16% and 99.18% (*M*= 96.57%, *SD*= 1.92%).

# Experiment 2

## Supplementary results

### Response accuracy.

The proportion of trials in which no answer was made before 20 s had passed ranged among participants from 0.00% to 13.06% (*M*= 5.44%*,* *SD* = 3.23%). A one-factorial repeated measures ANOVA indicated that the proportion of misses differed between context conditions**,** F*(3, 45) = 5.08,* p*= <.001,* *η*_G_^2^  = 0.163. Post-hoc paired-samples t-test revealed that the proportion of misses was higher in the *Novel – new locations* condition (*M*= 8.37%*,* *SD*= 4.86%) than in the *Repeated – 1 location* condition (*M* = 3.33%*,* *SD* = 5.99%), *t*(15) = ‑3.247, *p*= 0.005, *d*_z_ = 0.812, and higher than in the *Repeated – 2 location*s condition *M* = 3.54%*,* *SD* = 3.72%),  *t*(15) = -4.167, *p*= <0.001, *d*_z_ = 1.042. Bayesian *t*-Tests also delivered moderate to strong support that those pairs of conditions differed, *BF*_10_ = 9.111 and *BF*_10_ = 45.063. A possible explanation for this difference is that while each participant performed equally many trials of each condition, for each of the *repeated* context conditions only 6 scenes were used for 180 (6 × 30) trials, but 180 different scenes were used in the *novel* condition. This renders it more likely that there is a difficult scene among the novel trials than in the repeated scenes since if a scene is not too difficult to search through in one block it unlikely will be too difficult in another block. Simpler said: Because more different scenes are used for the novel context condition than for the repeated context conditions, the probability that there is a (too) difficult search display in the novel context condition is higher.

The proportion of response errors (e.g., “T” when the target was an “L”) did not significantly differ between *novel* (*M*= 3.30%*,* *SD*= 3.06%) and the *Repeated – 1 location* contexts (*M*= 2.15%*,* *SD*= 2.10%), *t*(15) = -1.633, *p*= 0.123, *d*_z_ = 0.408, *BF*_10_= 0. 764, or the *Repeated – 2 location*s contexts (*M*= 3.19%*,* *SD*= 4.29%), *t*(15) = -0.197, *p*= 0.846, *d*_z_ = 0.049, *BF*_10_= 0. 260.

Referring to all trials (no exclusions made, thus including not only false discrimination but also misses), accuracy ranged among participants between 85.28% and 96.11% (*M* = 91.50%, *SD* = 3.23%). When referring to all trials except those in which no answer was made within 20 s, accuracy ranged between 88.75% and 99.13% (*M*= 96.80, *SD*= 2.78).

### Sorting contextual cueing effects into minor and dominant for repeated-1-location contexts.

Following the logic in the analyses by Zellin et al. (2011), we also analyzed repeated-1-location contexts in a similar fashion to reveal whether the difference in contextual cueing effect between dominant and minor target locations might be an artefact of the sorting procedure: For each participant, the six contexts making up the repeated-1-location contexts, were randomly assigned to each other to build three pairs. For each pair, the context that showed a larger average contextual cueing effect was assigned to the “dominant” and the other to the “minor” category of pair dominance. As for the repeated-2-locations contexts, the contextual cueing effect for both categories were positive, however, this time, they both were significantly different from zero, too, *t*(15) = 12.509, *p*< .001,  *BF*_10_ = 4328618, *d*_z_ = 0.833, and *t*(15) = 4.184, *p*< .001,  *BF*_10_ = 46.482, *d*_z_ = 0.618. In a following step, we tested whether the effect for dominant and minor locations, respectively, differed between repeated-2-locations and repeated-1-location contexts. For dominant target locations, it was unclear whether the descriptively smaller contextual cueing effects in 2-location than in 1-locations repeated contexts, [830 vs. 1011 ms], can still be regarded as comparable, *t*(15) = 1.945, *p*= .071,  *BF*_10_ = 1.152, *d*_z_ = 0.486. The contextual cueing effect for the minor targets was significantly smaller in the 2-locations-repeated contexts than in the 1-location-repeated contexts, [121 vs. 401 ms], *t*(15) = 2.746, *p*= .015,  *BF*_10_ = 3.923, *d*_z_ = 0.687.

When only regarding the second half of the experiment, the pattern remained that the contextual cueing effect for the dominant location showed a trend to be smaller in contexts with two than one possible locations, *t*(15) = 2.469, *p*= .026,  *BF*_10_ = 2.512 *d*_z_ = 0.617, and for the minor location the effect was significantly smaller in contexts with two possible locations, *t*(15) = 3.900, *p*= .0001,  *BF*_10_ = 28.225, *d*_z_ = 0.975.

However, we argue that the comparison between the major and minor target locations in contexts with one or two target locations cannot be made without restrictions. It must be noted, that the sorting procedure for the repeated-1-location contexts yielded in half as many data points than for the repeated-2-location contexts, since two different scenes had to be matched to determine a dominant and a minor target. In that way, not only the varied mapping between context and target location, the perhaps more difficult learning, and a second already learnt (major) target location might aggravate finding the minor target. Instead, the lower level of the contextual cueing effect for the minor target location (but also for the major target location) might be expectable just because there were fewer instances to learn an association.

## Supplementary Tables

Supplementary Table 1

*Post-hoc one-way ANOVAs predicting manual response time in Experiment 2.*

|  | Group |  | | | | | |
| --- | --- | --- | --- | --- | --- | --- | --- |
| Predictor | Epoch | df_Num_ | df_Den_ | ε_GG_ | F | p | η_G_^2^ |
| Context | 1 | 3 | 45 |  | 0.80 | 0.476 | 0.025 |
|  | 2 | 3 | 45 |  | 16.19 | <.001 | 0.298 |
|  | 3 | 3 | 45 |  | 9.10 | <.001 | 0.199 |
|  | 4 | 3 | 45 |  | 15.01 | <.001 | 0.268 |
|  | 5 | 2.27 | 33.98 | 0.755 | 28.97 | <.001 | 0.442 |
|  | 6 | 3 | 45 |  | 34.46 | <.001 | 0.509 |
|  | Context |  |  |  |  |  |  |
| Epoch | Repeated-1-location | 1.62 | 24.27 | 17.05 | 1.62 | <.001 | 0.324 |
|  | Repeated-2-locations | 2.39 | 35.90 | 15.48 | 2.39 | <.001 | 0.189 |
|  | Repeated-1-hemifield | 5.00 | 75.00 | 8.15 | 5.00 | <.001 | 0.223 |
|  | Novel-new locations | 2.50 | 37.52 | 2.37 | 2.50 | 0.047 | 0.071 |

*Note*. *df*_Num_ indicated numerator degrees of freedom. *df*_Den_ indicates denominator degrees of freedom. ε_GG_ indicates the Greenhouse-Geisser multiplier for the degrees of freedom, p-values and degrees of freedom in the table incorporate this correction. η_G_^2^ indicates generalized eta-squared.

Supplementary Table 2

*Post-hoc t-tests of manual response time between the different repeated context conditions and the novel (new locations) context condition.*

| Epoch | Context compared to Novel-new locations | n_1_ | n_2_ | t | df | p |  |
| --- | --- | --- | --- | --- | --- | --- | --- |
| 1 | Repeated-1-location | 16 | 16 | –1.21 | 15 | 0.245 | ns |
|  | Repeated-2-locations | 16 | 16 | –0.79 | 15 | 0.442 | ns |
|  | Repeated-new locations | 16 | 16 | 0.17 | 15 | 0.863 | ns |
| 2 | Repeated-1-location | 16 | 16 | –5.39 | 15 | <.001 | *** |
|  | Repeated-2-locations | 16 | 16 | –3.94 | 15 | 0.001 | ** |
|  | Repeated-new locations | 16 | 16 | –0.84 | 15 | 0.413 | ns |
| 3 | Repeated-1-location | 16 | 16 | –4.99 | 15 | <.001 | *** |
|  | Repeated-2-locations | 16 | 16 | –1.59 | 15 | 0.133 | ns |
|  | Repeated-new locations | 16 | 16 | 0.95 | 15 | 0.358 | ns |
| 4 | Repeated-1-location | 16 | 16 | –5.09 | 15 | <.001 | *** |
|  | Repeated-2-locations | 16 | 16 | –5.16 | 15 | <.001 | *** |
|  | Repeated-new locations | 16 | 16 | –1.69 | 15 | 0.111 | ns |
| 5 | Repeated-1-location | 16 | 16 | –7.36 | 15 | <.001 | **** |
|  | Repeated-2-locations | 16 | 16 | –6.83 | 15 | <.001 | **** |
|  | Repeated-new locations | 16 | 16 | –2.16 | 15 | 0.047 | ns |
| 6 | Repeated-1-location | 16 | 16 | –10.77 | 15 | <.001 | **** |
|  | Repeated-2-locations | 16 | 16 | –7.05 | 15 | <.001 | **** |
|  | Repeated-new locations | 16 | 16 | –3.46 | 15 | 0.004 | * |

Supplementary Table 3

*T-tests of contextual cueing effect in manual response time by experiment half and dominance of target location.*

| Experiment half | Context condition | Dominance | n_1_ | n_2_ | t | df | p | *d*_z_ | *BF*_10_ |
| --- | --- | --- | --- | --- | --- | --- | --- | --- | --- |
| 1 | Repeated-1-location | Dominant | 16 | 16 | 9.183 | 15 | < .001 | 0.727 | 1.31×10^7^ |
|  |  | Minor | 16 | 16 | 0.283 | 15 | .781 | 0.276 | 1.52×10^0^ |
|  | Repeated-2-locations | Dominant | 16 | 16 | 5.773 | 15 | .004 | 0.714 | 5.44×10^4^ |
|  |  | Minor | 16 | 16 | -0.947 | 15 | .358 | -0.127 | 2.40×1^1^ |
| 2 | Repeated-1-location | Dominant | 16 | 16 | 8.805 | 15 | < .001 | 1.290 | 7.71×10^6^ |
|  |  | Minor | 16 | 16 | 5.846 | 15 | < .001 | 1.173 | 6.22×10^4^ |
|  | Repeated-2-locations | Dominant | 16 | 16 | 11.495 | 15 | < .001 | 1.159 | 2.58×10^8^ |
|  |  | Minor | 16 | 16 | 5.082 | 15 | < .001 | 0.410 | 1.48×10^4^ |

*Note*. The *t* statistic refers to paired *t*-tests between the mentioned context condition and condition of novel contexts. For the context of novel conditions, no separation between dominant and minor locations was made so all data points from the first or second experiment half respectively were used unrespetively whether compared to dominant/minor locations. The reported Bayes Factor *BF*_10_ tests the hypothesis that δ >= 0 against the hypothesis that δ < 0.

Supplementary Table 4

*Post-hoc t-tests of number of searching fixations between the different repeated context conditions and the novel (new locations) context condition.*

| Epoch | Context compared to Novel-new locations | n_1_ | n_2_ | t | df | p |
| --- | --- | --- | --- | --- | --- | --- |
| 1 | Repeated-1-location | 16 | 16 | –1.61 | 15 | .128 |
|  | Repeated-2-locations | 16 | 16 | –1.1 | 15 | .288 |
|  | Repeated-1-hemifield | 16 | 16 | –0.05 | 15 | .958 |
| 2 | Repeated-1-location | 16 | 16 | –5.52 | 15 | <.001 |
|  | Repeated-2-locations | 16 | 16 | –4.13 | 15 | .001 |
|  | Repeated-1-hemifield | 16 | 16 | –0.96 | 15 | 0.351 |
| 3 | Repeated-1-location | 16 | 16 | –4.79 | 15 | <.001 |
|  | Repeated-2-locations | 16 | 16 | –1.99 | 15 | 0.065 |
|  | Repeated-1-hemifield | 16 | 16 | 0.99 | 15 | 0.338 |
| 4 | Repeated-1-location | 16 | 16 | –5.63 | 15 | <.001 |
|  | Repeated-1-hemifield | 16 | 16 | –6.07 | 15 | <.001 |
|  | Repeated-new locations | 16 | 16 | –2.19 | 15 | 0.045 |
| 5 | Repeated-1-location | 16 | 16 | –6.85 | 15 | <.001 |
|  | Repeated-2-locations | 16 | 16 | –5.85 | 15 | <.001 |
|  | Repeated-new locations | 16 | 16 | –2.00 | 15 | 0.064 |
| 6 | Repeated-1-location | 16 | 16 | –10.67 | 15 | <.001 |
|  | Repeated-2-locations | 16 | 16 | –6.61 | 15 | <.001 |
|  | Repeated-new locations | 16 | 16 | –2.65 | 15 | 0.018 |

## Supplementary Figures


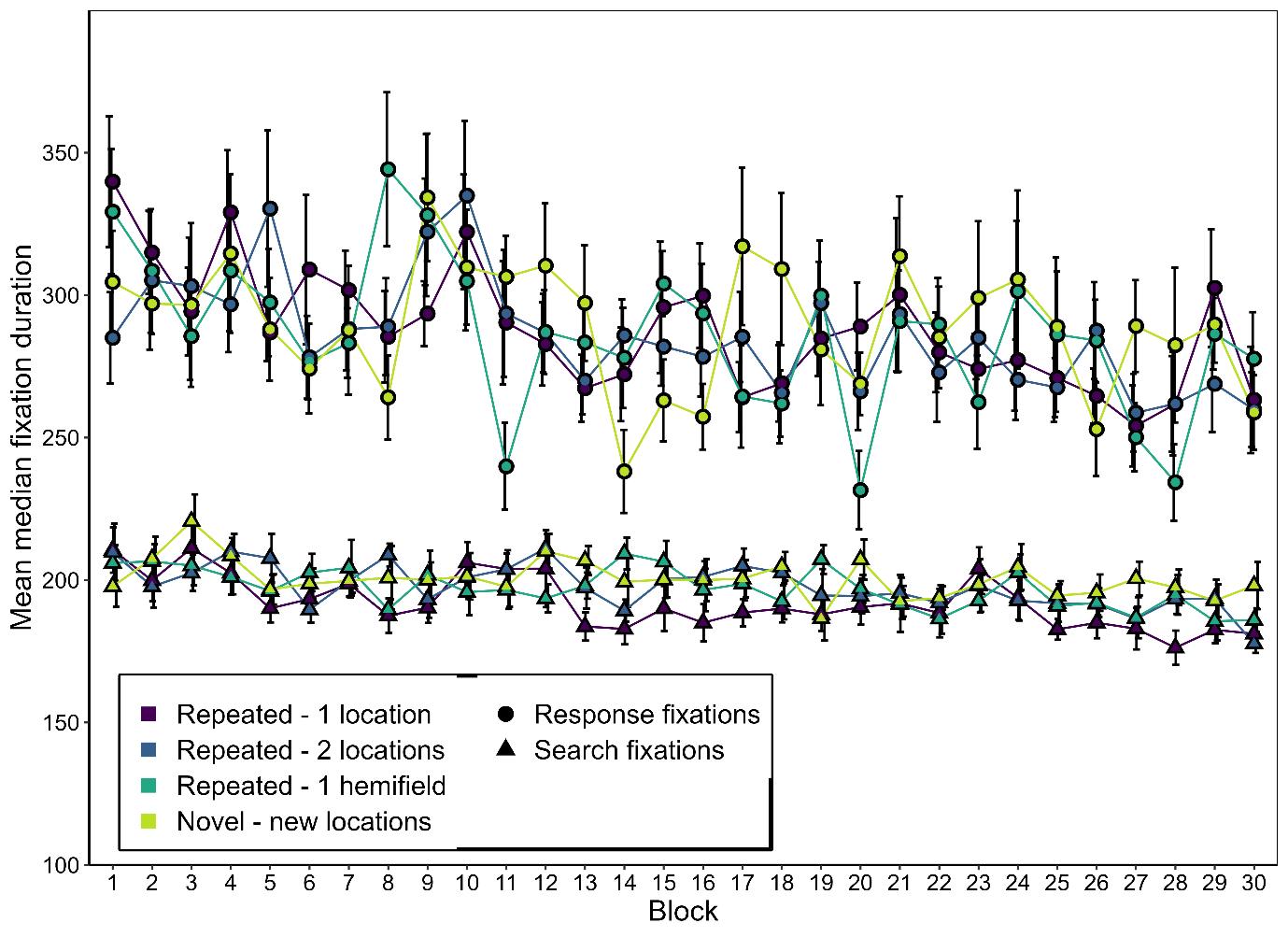


**Supplementary Figure 1.** Duration of searching and responding fixations as a function of block and context in Experiment 1. Error-bars represent standard errors for within-subjects designs that were calculated considering each fixation type separately (Morey, 2008).
